# Hyaluronic acid synthesis, degradation, and crosslinking in equine osteoarthritis: TNF-α-TSG-6-mediated HC-HA formation

**DOI:** 10.1186/s13075-021-02588-7

**Published:** 2021-08-20

**Authors:** Diana C. Fasanello, Jin Su, Siyu Deng, Rose Yin, Marshall J. Colville, Joshua M. Berenson, Carolyn M. Kelly, Heather Freer, Alicia Rollins, Bettina Wagner, Felipe Rivas, Adam R. Hall, Elaheh Rahbar, Paul L. DeAngelis, Matthew J. Paszek, Heidi L. Reesink

**Affiliations:** 1 Department of Clinical Sciences, College of Veterinary Medicine, Cornell University, Ithaca, NY USA; 2 Robert Frederick Smith School of Chemical and Biomolecular Engineering, Cornell University, Ithaca, NY USA; 3 Department of Molecular Medicine, College of Veterinary Medicine, Cornell University, Ithaca, NY USA; 4 Department of Population Medicine and Diagnostic Sciences, College of Veterinary Medicine, Cornell University, Ithaca, NY USA; 5 Virginia Tech-Wake Forest University School of Biomedical Engineering and Sciences, Wake Forest School of Medicine, Winston-Salem, NC USA; 6 Department of Biochemistry & Molecular Biology, University of Oklahoma Health Sciences Center, Oklahoma City, OK USA

**Keywords:** Synovial fluid, Viscosity, Microrheology, SEC-MALS, Heavy chain-hyaluronic acid, Cartilage, Synovial membrane

## Abstract

**Background:**

TNF-α-stimulated gene 6 (TSG-6) protein, a TNF-α-responsive hyaladherin, possesses enzymatic activity that can catalyze covalent crosslinks of the polysaccharide hyaluronic acid (HA) to another protein to form heavy chain-hyaluronic acid (HC-HA) complexes in pathological conditions such as osteoarthritis (OA). Here, we examined HA synthase and inflammatory gene expression; synovial fluid HA, TNF-α, and viscosity; and TSG-6-mediated HC-HA complex formation in an equine OA model. The objectives of this study were to (1) evaluate the TNF-α-TSG-6-HC-HA signaling pathway across multiple joint tissues, including synovial membrane, cartilage, and synovial fluid, and (2) determine the impact of OA on synovial fluid composition and biophysical properties.

**Methods:**

HA and inflammatory cytokine concentrations (TNF-α, IL-1β, CCL2, 3, 5, and 11) were analyzed in synovial fluid from 63 OA and 25 control joints, and HA synthase (*HAS1-3*), *TSG-6*, and hyaluronan-degrading enzyme (*HYAL2*, *HEXA*) gene expression was measured in synovial membrane and cartilage. HA molecular weight (MW) distributions were determined using agarose gel electrophoresis and solid-state nanopore measurements, and HC-HA complex formation was detected via immunoblotting and immunofluorescence. SEC-MALS was used to evaluate TSG-6-mediated HA crosslinking, and synovial fluid and HA solution viscosities were analyzed using multiple particle-tracking microrheology and microfluidic measurements, respectively.

**Results:**

TNF-α concentrations were greater in OA synovial fluid, and *TSG6* expression was upregulated in OA synovial membrane and cartilage. TSG-6-mediated HC-HA complex formation was greater in OA synovial fluid and tissues than controls, and HC-HA was localized to both synovial membrane and superficial zone chondrocytes in OA joints. SEC-MALS demonstrated macromolecular aggregation of low MW HA in the presence of TSG-6 and inter-α-inhibitor with concurrent increases in viscosity.

**Conclusions:**

Synovial fluid TNF-α concentrations, synovial membrane and cartilage *TSG6* gene expression, and HC-HA complex formation were increased in equine OA. Despite the ability of TSG-6 to induce macromolecular aggregation of low MW HA with resultant increases in the viscosity of low MW HA solutions in vitro, HA concentration was the primary determinant of synovial fluid viscosity rather than HA MW or HC-HA crosslinking. The TNF-α-TSG-6-HC-HA pathway may represent a potential therapeutic target in OA.

**Supplementary Information:**

The online version contains supplementary material available at 10.1186/s13075-021-02588-7.

## Background

Musculoskeletal trauma and sport-related injuries, including intra-articular fractures, dislocations, and ligament, meniscal, and joint capsule tears, are frequently associated with the development of post-traumatic osteoarthritis (PTOA) [1]. PTOA also results in inflammation of joint tissues, including the synovium, cartilage, subchondral bone, and surrounding soft tissues [2, 3] following induction of the pro-inflammatory OA “master regulators” tumor necrosis factor-α (TNF-α) and interleukin-1β (IL-1β) [4]. Synovial fluid TNF-α has been associated with radiographic OA progression in human knee OA [5], and increased synovial fluid TNF-α has been reported in both naturally occurring equine carpal OA [6] and in an experimentally induced equine cartilage carpal defect model [7].

Hyaluronic acid (HA) confers viscoelastic properties to synovial fluid and, in concert with lubricin/proteoglycan 4, enables low-friction lubrication of articular cartilage [8, 9]. HA concentrations have been reported to decrease or remain unchanged in OA [10, 11], with HA concentration and size more strongly associated with age than OA severity in human knee OA [10]. In osteoarthritic human knee joints, HA concentration decreases [10], with a corresponding reduction in molecular weight (MW), resulting in a larger proportion of low molecular weight, pro-inflammatory HA [12, 13]. Besides variation in HA MW, there are few post-synthetic modifications that occur to HA. One of these unique modifications is the covalent crosslinking of HA monomers by the TNF-α-inducible protein TNF-α-stimulated gene 6 protein (TSG-6) [14, 15]. TSG-6 is a multifunctional hyaladherin protein induced by the inflammatory cytokines TNF-α and IL-1β, with both pro- and anti-inflammatory properties depending upon the tissue type and context [16]. TSG-6 can bind non-covalently and reversibly to HA through its Link domain, resulting in the crosslinking of HA chains [14, 17]; TSG-6 can also induce a covalent modification of HA by facilitating the transfer of heavy chains (HC) via a trans-esterification reaction [18] from the proteoglycan inter-alpha trypsin inhibitor (IαI), a protein found in serum and pathological synovial fluid [19].

TSG-6-induced HC-HA complex formation occurs in diverse tissue types, including mammalian cumulus cells, and endothelial, epithelial, and smooth muscle cells in the gastrointestinal tract [20, 21]. HC-HA complex formation is critical to mammalian female fertility, with HC crosslinking of HA resulting in a stabilized and mucified matrix of the cumulus cell-oocyte complex (COC) [22]. In irritable bowel disease, TNF-α-induced inflammation results in the production of large HC-HA complexes, or cables, along smooth muscle and endothelial cells in the bowel which present a persistent inflammatory insult to the local environment, promoting immune cell recruitment [21]. In the joint, TSG-6 is reported to have dual activities: reducing cartilage matrix assembly through impaired HA binding to HA binding protein and aggrecan while also inhibiting plasmin activity, thereby limiting matrix metalloprotease activation [23]. In murine collagenase-induced arthritis models, treatment with recombinant TSG-6 resulted in ectopic bone formation in one study [24], but decreased disease incidence and footpad swelling and erythema in another [25]. Constitutive overexpression of TSG-6 resulted in chondroprotective but not anti-inflammatory effects in transgenic mice with antigen-induced arthritis [26].

Increased synovial fluid TSG-6 levels have been detected in human OA patients [27], and HC-HA complex formation has been observed in the synovial fluid of RA patients using protein sequencing and mass spectrometry [28]. However, the effects of TSG-6 and HC-HA complex formation on the biochemical and biophysical properties of synovial fluid and their tissue distributions in the osteoarthritic joint are not well understood. In humans, synovial fluid TSG-6 is a biomarker for OA progression, with a significant correlation between TSG-6 activity and radiographic OA progression in the knee over a 3-year period [29]. However, it is unclear whether TSG-6 expression in the joint is functioning to resolve or perpetuate inflammation and what effects TSG-6-mediated HC-HA crosslinking have on joint tissue and synovial fluid properties.

Here, we explore the TNF-α-TSG-6-HC-HA axis in a naturally occurring equine model of carpal PTOA secondary to intra-articular fracture. We hypothesized that HA concentrations and HA MW distributions would be decreased in OA joints and that upregulation of the TNF-α-TSG-6-HC-HA signaling axis would result in alterations to synovial fluid composition and viscosity.

## Methods

### Equine population and sample collection

This prospective case-control study included case horses (*n* = 63) presenting to the Cornell University Equine Hospital for arthroscopic evaluation and treatment of carpal osteochondral fragmentation and/or osteoarthritis (OA) affecting either the antebrachiocarpal joint (ACJ, *n* = 16), middle carpal joint (MCJ, *n* = 36), or both joints (*n* = 11). Seven horses were donated for euthanasia and tissue collection due to severe carpal OA (ACJ, *n* = 2 and MCJ, *n* = 5). Control horses (*n* = 25) included horses enrolled in other research projects unrelated to carpal disease that were euthanized due to their inclusion in other terminal research projects. All joints were evaluated with gross examination at necropsy. Exclusion criteria included carpal joint sepsis or a known history of intra-articular medication in the month prior to presentation. In order to avoid non-independence of samples, one joint was randomly chosen and reported per horse in cases where samples were available from multiple joints. An average of 4.9 ± 2.4 mL of synovial fluid was collected per carpal joint at a single time point (i.e., arthroscopy or euthanasia). Synovial fluid samples were collected from 87 study horses; however, synovial membrane and cartilage tissues were only available from a subset of horses (*n* = 36 and 18, respectively) obtained via either arthroscopic biopsy or postmortem dissection (Table S1). HA ELISAs, gel electrophoresis, and synovial fluid cytokine measurements were performed for the majority of synovial fluid samples, whereas subsets of randomly selected synovial fluid samples were chosen for SS-nanopore measurements (*n =* 24) and multiple particle-tracking microrheology (MPTM) measurements (*n* = 35). The complete study population consisted of 53 females, 22 castrated males, and 13 intact males with a median age of 4 years (range 1 to 22 years). The healthy case population included 14 females, 8 castrated males, and 3 intact males with a median age of 5 years (range from 2 to 11 years); breeds included 14 Thoroughbreds, 7 mixed light breeds, 3 Standardbreds, and 1 Quarter Horse.

Cases were scored on the basis of severity of carpal OA as healthy (0), mild OA (1), moderate OA (2), or severe OA (3), using a previously described scale that included radiographic assessment with or without additional corroboration with arthroscopic findings [6, 30]. The study population consisted predominantly of racing breeds, including Thoroughbreds (*n* = 62) and Standardbreds (*n* = 12), in addition to Quarter Horses (*n* = 7) and mixed light breeds (*n* = 7). Cartilage and/or osteochondral pathology was detected for the following bones: radial carpal bone (*n* = 37), third carpal bone (*n* = 21), radius (*n* = 15), intermediate carpal bone (*n* = 12), second carpal bone (*n* = 4), ulnar carpal bone (*n* = 2), fourth carpal bone (*n* = 2), and accessory carpal bone (*n* = 1). In 25 cases, pathology of multiple bones was detected within each joint, and there were 9 cases where the specific location of pathology was not recorded. The 63 OA horses included 39 females, 14 castrated males, and 10 intact males and had a median age of 3 years (range 1–22 years); breeds included 48 Thoroughbreds, 9 Standardbreds, and 6 Quarter Horses. All animal and tissue harvesting protocols were approved by Cornell University’s Institutional Animal Care and Use Committee.

### Hyaluronan (HA) ELISAs

Two commercially available ELISAs were used in parallel to quantify HA concentrations in carpal synovial fluid (SF) (*n* = 25 healthy, *n* = 61 OA), including a sandwich ELISA (HA DuoSet®, R&D Systems, Minneapolis, MN) and a competitive ELISA (Echelon Biosciences Inc., Salt Lake City, UT). This dual concentration analysis was employed as a precaution to help prevent bias of HA MW sensitivity in the assay formats. For the sandwich ELISA, synovial fluid was diluted 1:80,000 with phosphate-buffered saline (PBS), and HA concentration was measured using recombinant human aggrecan (rhAggrecan) as the capture reagent and biotinylated rhAggrecan as the detection reagent. HA concentrations were also measured using a competitive ELISA following proteinase K (PK) digestion of synovial fluid and ethanol precipitation of HA as previously reported [31]. For PK digestion, 10 μL of SF was added to 190 μL of PBS buffer and digested with 20 μg of PK at 60°C for 2 h. The HA in the PK-digested mix was precipitated by adding four volumes of ethanol, followed by incubation at −20°C overnight. Following centrifugation at 14,000×*g* for 10 min, the HA precipitate was dissolved in 200 μL of PBS buffer (the resulting HA concentration in PBS was diluted 1:20). After an additional 100-fold dilution in Echelon diluent (final 2000-fold dilution), the PK-digested and ethanol-precipitated HA was determined following the manufacturer’s instructions.

### HA agarose gel electrophoresis for determination of HA MW

HA MW distributions were determined for synovial fluid samples (*n* = 25 healthy, *n* = 61 OA) by performing agarose gel electrophoresis and Stains-All detection. HA agarose gel electrophoresis was performed as previously described [32] with slight modification. SF samples were first diluted (1:15) in PBS and incubated with PK at 37°C overnight. PK-digested samples were loaded onto 0.5% agarose gels in Tris-acetate-EDTA (TAE) buffer and run at 55 V for 8 h along with Select-HA HiLadder and Select-HA Mega Ladder (Hyalose LLC, Oklahoma City, OK). The gel was stained with 0.005% Stains-All (Millipore Sigma, Burlington, MA) in 50% ethanol for 16 h and then de-stained with 10% ethanol. Densitometric scanning of stained gels was used to detect relative HA content and molecular weight distribution in synovial fluid. Images were captured with a ChemiDoc Imaging System (Bio-Rad, Hercules, CA). Gel image analysis was performed as described in Cowman et al., with some modification [33]. Gel images were opened in Fiji, and a rolling ball technique was used to subtract background in order to standardize measurements. A calibration curve was generated for each gel by correlating the molecular weight of each standard to the migration distance in pixels. Densitometric profiles were generated for each sample, and the calibration curve was applied to calculate the relative absorbance of the sample. Finally, weight average (*M*_*w*_), number average (*M*_*n*_), and polydispersity index (PDI = *M*_*w*_/*M*_*n*_) were calculated using provided equations.

### Solid state (SS)-nanopore analysis of HA

For SS-nanopore analysis of HA MW distributions in a subset of SF samples (*n* = 6 healthy, *n* = 18 OA), HA was isolated from each synovial fluid sample using an approach described previously [34]. Briefly, 50 μL of synovial fluid was incubated with 1.8 U/mL PK (New England Biolabs, Ipswich, MA) for 15 min at 37 °C to digest protein components including any bound to HA. An equal volume of a phenol:chloroform:isoamyl alcohol (25:24:1 v/v/v, Fisher Scientific) was then added to the sample and mixed by vortexing before being centrifuged for 15 min at 14,000×*g* in a Phase Lock Gel Tube (QuantaBio, Beverly, MA) to separate the aqueous components (including HA) from the other organics. The process was repeated using pure chloroform to remove residual phenol from the aqueous phase. To isolate pure HA, 150 μL of streptavidin-conjugated magnetic beads (10 mg/mL, Dynabeads M-280, Invitrogen, Carlsbad, CA) was washed by adding buffer, mixing gently, and aspirating under magnetic field. This washing process was performed three times with 1X PBS and 0.05% Tween and then three times with 1X PBS only. After washing, packed beads were resuspended in 50 μL of 1X PBS, and 21 μL of biotinylated versican G1 domain (1.23 μg/μL, Echelon Biosciences, Salt Lake City, UT) was added directly to the beads, mixed, and incubated for 1 h at room temperature on a rocker. After incubation, the beads were washed three times in 1X PBS to remove unbound protein. Aqueous isolate from the phenol-chloroform extraction was added to the packed versican-streptavidin beads to resuspend them and the solution was mixed and left to incubate at room temperature for 24 h with gentle rocking. The sample was placed on a magnet to pull down the beads (with bound HA) and the supernatant was aspirated. The beads were washed three times with 1X PBS and deionized water was added to the sample to a final volume of 50 μL. To denature the bVG1 and release the bound HA, the sample was placed on a heating block at 95 °C for 15 min. Finally, the vial was placed on a magnet and the solution containing released, purified HA was removed and stored at −20 °C until use.

The isolated HA (12 μL with 5–15 ng HA) was loaded into a grounded flow cell chamber (*cis-*) and the amplifier was used to apply a positive voltage to the opposite (*trans-*) chamber. Data was collected at a rate of 200 kHz with a four-pole Bessel filter designed to be 100 kHz (empirically determine to be 57 kHz). Analysis was performed using custom software (LabView, National Instruments, Austin, TX). A 5-kHz low-pass filter was applied to all data, and event thresholds were defined as a deviation of at least five standard deviations (5*σ*) from baseline current with a duration between 25 μs and 2.5 ms. ECD was calculated for each event as the area defined by the deviation [35] determined by integrating measured current for the time it remained above the threshold value. The calculated ECD value for each event was converted to MW by comparison to a calibration produced through identical nanopore measurements of 7 quasi-monodisperse HA samples (Hyalose, Oklahoma City, OK) having MWs of 54 kDa, 81 kDa, 130 kDa, 237 kDa, 545 kDa, 1076 kDa, and 2384 kDa, respectively, each having a MW distribution within 5% of the reported mean (polydispersity = 1.001–1.035, as estimated by multi-angle light scattering with size exclusion chromatography).

### Multiple particle-tracking microrheology (MPTM) measurements

Multiple particle-tracking microrheology [36] was used to quantify SF viscosity in a subset of samples (*n* = 18 healthy, *n* = 17 OA). In brief, 15 μL of synovial fluid was loaded with 0.5-nm yellow-green fluorescent beads (FluoSpheres™ Carboxylate-Modified Microspheres, 0.5 μm, yellow-green fluorescent) and imaged on a custom-built instrument consisting of an inverted microscope (IX71, Olympus) equipped with a 60× NA 1.2 water-immersion objective lens and 1.6× optical coupler. Fluorescence excitation was generated by a 488-nm laser (Sapphire-LP, Coherent) expanded 8.3× before focusing on the objective back aperture by a 300-mm tube lens (ThorLabs). Fluorescence emission was imaged with an EMCCD (897 Ultra, Andor) through a standard FITC filter set (Chroma) using the Micro-Manager software package (Open Imaging). Three 30-s videos were taken per sample with several locations in each sample targeted to collect particle movement data. Each video contains approximately 15 particles in the frame. Analysis of motion was performed using the Trackpy Python package, and images were acquired at 16 Hz.

### qRT-PCR for HA-associated gene expression

Synovial membrane tissue was collected from a subset of horses (*n* = 11 healthy, *n* = 25 OA), and cartilage tissue was only available from 4 healthy and 14 OA joints. Gene expression for enzymes and proteins involved in HA synthesis, degradation, and stability were quantified in synovial membrane and cartilage, including the hyaluronan synthases (*HAS1*, *HAS2*, and *HAS3*), TNF-α-stimulated gene 6 protein (*TSG6*), hyaluronidase II (*HYAL2*), and hexosaminidase subunit α (*HEXA*). Total RNA was extracted from synovial membrane (SM) and cartilage tissues. Synovial membrane RNA was extracted and purified using the E.Z.N.A tissue kit (Omega Bio-Tek, Norcross, GA). Cartilage RNA was isolated and purified using the RNeasy lipid tissue kit (QIAGEN, Gaithersburg, MD). The extracted RNA was subjected to DNase I digestion on-column to remove any genomic DNA. RNA concentrations and quality were determined using 16-well NanoQuant plates and a SPARK 10M microplate reader (TECAN, Zürich, Switzerland) (Table S2).

Gene expression was detected by quantitative real-time polymerase chain reaction (qRT-PCR) using the ABI PRISM 7900 sequence detection system (Applied Biosystems, Foster City, CA). All samples were analyzed in duplicate using the Power SYBR green RNA-to-C_T_ one-step kit (Applied Biosystem Inc., Carlsbad, CA). Primers (Table 1) were designed using NCBI Primer3-Blast or Lasergene (DNASTAR, Madison, WI). A qRT-PCR checklist containing additional details is included in Table S2.

**Table 1:** Genes, accession numbers, primer sequences, and amplicon sizes for qRT-PCR

Genes	Accession number	Primer sequences	Amplicon size (bp)
*HAS1*	XM_023650323.1	For: GCGATACTGGGTGGCCTTCAATGT Rev: CTGTATAGGCCTAGGGGACCACTG	90
*HAS2*	NM_001081801.2	For: GGCCGGTCGTCTCAAATTCA Rev: TCACAATGCATCTTGTTCAGCTC	132
*HAS3*	XM_023637194.1	For: CGTGGGCGCATCTGGAACATT Rev: CTCTGCATTGCCCCGAAGGAAG	99
*TSG6*	NM_001081906.1	For: ATCCTGAGCAGCCCCTAACA Rev: TTGAATCCCCATCCGTGAGC	108
*HYAL2*	XM_014731656.1	For: CTCACAGGGCTTAGCGAGAT Rev: GGTACTGGCAGGTCTCCGTG	124
*HEXA*	XM_001494311.4	For: AAGGAGCTGGAACTGGTCAC Rev: TCAGGGGTACCGTCAAATGC	137
*18S* rRNA	NR_046271.1	For: GGCGTCCCCCAACTTCTT Rev: AGGGCATCACAGACCTGTTATTG	77

In brief, 30 ng of synovial membrane RNA was added in a total of 20-μL reaction mix containing the SYBR RT-PCR mix and RT enzyme mix. For cartilage RNA, 15 ng of the total RNA was used in a total of 10-μL reaction mix. Both synovial membrane and cartilage samples were prepared in duplicate. The qRT-PCR was run at 48°C for 30 min and 95°C for 10 min, followed by 40 cycles of 95°C/15 s and 60°C/1 min. Successful qRT-PCR was verified by both analysis of dissociation curves and agarose gel electrophoresis. All values were normalized to the housekeeping gene 18S rRNA. Relative gene expression was analyzed using the 2^−∆∆*C*T^ method, where ∆*C*_T_ = *C*_T_ (gene of interest) – *C*_T_ (18S rRNA), ∆∆*C*_T_ = (individual ∆*C*_T_) – healthy ∆*C*_T_ average and calculated as 2^−∆∆*C*T^.

### Chemokine multiplex assay

This chemokine multiplex assay was validated at the Animal Health Diagnostic Center at Cornell University and was performed for a subset of SF samples (*n* = 20 healthy, *n* = 54 OA). The fluorescent bead-based assay quantifies six cytokines/chemokines (IL-1β, TNF-α, CCL2, CCL3, CCL5, and CCL11) using pairs of monoclonal antibodies (mAbs). The procedures of coupling mAbs to fluorescent beads (Luminex Corp., Austin, TX, USA) and performing the remainder of the assay were previously described in detail for other cytokines [37] and were identical for this assay. The following beads and mAbs were coupled: bead 33 with TNF-α mAb 292-1, bead 34 with CCL11 mAb 24, bead 35 with IL-1β mAb 84-2, bead 36 with CCL5 mAb 91-1, bead 37 with CCL2 mAb 104-2, and bead 42 with CCL3 mAb 77-2. Specificity of the mABs to their respective chemokines and recognition of native proteins were confirmed for all mAbs [38] before use in the assay.

The six recombinant proteins were expressed in mammalian cells as IL-4 fusion proteins [38, 39]. A mixture of the six recombinant chemokines was included in different concentrations (5-fold dilutions in PBS with 1% (w/v) bovine serum albumin (BSA) and 0.05% (w/v) sodium azide (blocking buffer)) to create standard curves for chemokine concentration quantification in samples. Synovial fluid samples were diluted 1:2 in blocking buffer. Millipore Multiscreen HTS plates (Millipore, Danvers, MA) were soaked with PBS with 0.1% (w/v) BSA, 0.02% (v/v) Tween 20, and 0.05% (w/v) sodium azide (PBS-T) using a ELx50 plate washer (Biotek Instruments Inc., Winooski, VT) for 2 min. The blocking solution was removed from the plates and 50 μL of standards or samples was added. The bead solution (50 μL), containing 5 × 10^3^ beads per bead number, was added to each well and incubated for 30 min on a shaker at room temperature. The plates were then washed with PBS-T. The detection antibody mixture (50 μL) that was diluted in blocking buffer was added to each well and incubated for an additional 30 min on a shaker at room temperature. These mixtures included six biotinylated mAbs: TNF-α mAb 48-1, CCL11 mAb 25, IL-1β mAb 62-7, CCL5 mAb 46-1, CCL2 mAb 49, and CCL3 mAb 289-2 [38]. Plates were washed again and 50 μL of streptavidin-phycoerythrin (Invitrogen, Carlsbad, CA) was added to the plates prior to another 30-min incubation period as above. Plates were then washed for a final time, beads were resuspended in 100 μL of blocking buffer, and the plates were placed on the shaker for an additional 15 min. The plate was analyzed in a Luminex 200 instrument (Luminex Corp., Austin, TX, USA). The data were reported as median fluorescent intensities. The logistic 5p formula (*y* = *a* + *b*/(1 + (*x*/*c*)ˆ*d*)ˆ*f*) was used for standard curve fitting and calculation of chemokine concentrations (Luminex 200 Integrated System). Chemokine concentrations were reported in pg/mL. Because IL-1β and CCL3 concentrations were undetectable in the majority of synovial fluid samples, only descriptive statistics were reported.

### Detection of heavy chain-HA complex (HC-HA) via immunoblotting

Heavy chain-HA (HC-HA) was detected via immunoblotting (*n* = 25 healthy, *n* = 61 OA). Synovial fluid was digested with *Streptomyces* hyaluronidase (Millipore Sigma, Burlington, MA) to release HA-bound HC as described by Lauer et al. [40], with some modification. Briefly, 2 μL of synovial fluid was diluted 1:10 in PBS to achieve a final volume of 20 μL, and either 4 μL (0.2 U/μL) of hyaluronidase or 4 μL of PBS was added to the SF and incubated at 37°C for 2 h. The hyaluronidase-released HC in 12-μL reaction mix was detected by western blot probed with a rabbit polyclonal antibody against human inter-α-inhibitor (IαI) (Dako North America, Carpinteria, CA) and a secondary donkey anti-rabbit IgG-HRP (GE Healthcare, Chicago, IL). Densitometric quantification of the HC band on immunoblots was performed using ImageJ. Because HC-HA cannot readily enter into the SDS-PAGE gel due to the relative immobility of the large HC-HA complex, SF samples were digested with hyaluronidase (HAase) to release the HA-bound HC prior to gel electrophoresis. HA-bound HC was quantified by subtracting the HC band in undigested synovial fluid from the HC band in the HAase-digested SF. In order to normalize the two HC levels, the ratio of HC values divided by Pre-IαI was used for each sample. The HC-HA relative absorbance unit (a.u.) was defined as [HC (HAase-digested lane “+”)/Pre-IαI]–[HC (undigested synovial fluid lane “–”)/Pre-IαI] (Fig. 5A).

### Immunofluorescence of HC-HA in synovial membrane and cartilage

Immunofluorescence and confocal imaging of synovial membrane and cartilage tissues was performed as reported by Lauer et al. [15]. Deparaffinized and rehydrated synovial membrane or cartilage sections were blocked in 1% BSA in PBS at room temperature (RT) for 30 min. HC-HA complex was detected with the Dako anti-IαI antibody (courtesy of Vince Hascall), and HA was probed with a biotinylated hyaluronan binding protein (HABP) (Millipore Sigma, Burlington, MA). Both primary antibodies were simultaneously added to 1% BSA blocking buffer at a 1:100 dilution for the Dako anti-IαI antibody and 5 μg/mL final concentration of biotinylated HABP, followed by incubation at RT for 45 min. HC-HA complex was detected using Alexa Fluor 568-conjugated donkey anti-rabbit IgG (Thermo Fisher Sci., Waltham, MA) at 3 μg/mL, and HA was detected using Alexa Fluor 488-streptavidin at 1:500 in 1% BSA after incubation at RT for 1 h. Vectashield fluorescent mounting medium with DAPI (Vector Laboratories, Burlingame, CA) was used to prepare slides for microscopy. Confocal images were obtained with a 20× N.A. 0.8 air objective on a Zeiss LSM800 using Zen software (blue edition) for acquisition and post-processing. HC-HA immunofluorescence staining was scored from 0 to 4 where 0 = no staining, 1 = weak staining, 2 = weak-moderate staining, 3 = moderate-strong staining, and 4 = strong staining using 2 separate IF images by a single blinded observer. Insufficient cartilage tissues were available for scoring analysis.

### Size exclusion chromatography with multi-angle light scattering (SEC-MALS)

Size exclusion chromatography with multi-angle light scattering (SEC-MALS) was used to analyze the interaction of TSG-6 with HA and IαI in vitro. The SEC-MALS system includes a HPLC System with UV detector (Agilent Technologies, Santa Clara, CA), a static 18-angle light scattering detector unit (Dawn Heleos–II), and a refractive index detector (Optilab T-rEX). Three different solutions (1 experimental reaction plus 2 controls) were prepared in a total volume of 125 μL in PBS. The experimental reaction mix contained 4.5 μL of 150 kDa HA (Select-HA, Amsbio LLC, Cambridge, MA, 10 μg/μL), 33 μL of recombinant human TSG-6 (rhTSG6, R&D Systems, Cambridge, MN, 0.3 μg/μL), 37 μL of human plasma-derived IαI protein (IαIp, Athens Research & Technology, Athens, GA, 0.9 μg/μL), and 50.5 μL of PBS. The 2 control reactions were prepared by excluding either 4.5 μL of 150 kDa HA or 33 μL of TSG-6 based on the formula of the same experimental reaction mix. The reaction mix was incubated at room temperature for 1 h followed by incubation on ice for 4 h prior to being loaded onto a Superdex 200 Increase 10/300 GL column (GE Life Sci., Pittsburgh, PA) eluted in DPBS at 0.6 mL/min. Data were analyzed and molecular weights were determined using a *dn/dc* value of 0.185 mL/g for protein and 0.165 mL/g for HA with ASTRA 6.1 software (WYATT Technology, Santa Barbara, CA). Monomeric bovine serum albumin (Millipore Sigma, Burlington, MA) was used as a standard to normalize light scattering signal across detectors. GraphPad Prism 7.04 (GraphPad Software, San Diego, CA) was then used to convert axis units and adjust graphs.

### Viscosity measurements for TSG6-mediated HC-HA crosslinking reaction mixtures

To measure the viscosity of monodisperse HA solutions with and without TSG-6, 50 kDa Select-HA (Hyalose LLC, Austin, TX), human IαI protein (Athens Research & Technology, Athens, GA), and rhTSG6 were added, both alone and in combination, to DPBS containing 1 mM MgCl_2_ and incubated at 37°C for 4 h. The weight concentrations for each component are listed in Table 2. The reactions were terminated by adding EDTA to a final concentration of 10 mM. The viscosity of each reaction mix was measured using a m-VROC viscometer microfluidic device with an A05 chip (RheoSense Inc., San Ramon, CA) at 22°C using a Thermo CUBE (Solid State Cooling Systems, Wappingers Falls, NY). The shear rate and viscosity data were analyzed using mVROC Control v3.1.5 software. For each reaction mixture, 50 μL of solution was loaded using a 100-μL syringe, and the viscosity (cP) was measured using 4 different flow rates (50, 75, 100, 125 μL/min) with a total of 7 test segments (2 × 50, 2 × 75, 2 × 100, 1 × 125 μL/min). The equivalent apparent shear rates for the 4 flow rates were 2 × 950, 2 × 1420, 2 × 1890, and 1 × 2360 S^−1^, respectively. The data recorded from the shear rate 950 S^−1^ were excluded due to excessive variability. Therefore, a total of 5 technical replicates (from the apparent shear rates 2 × 1420, 2 × 1890, 1 × 2360 S^−1^) were included for data analysis.

**Table 2:** Components of TSG6-mediated HC-HA crosslinking reaction mixtures

Reaction	HA 50 kDa (10 mg/mL)	IαI (877 μg/mL)	TSG6 (300 μg/mL)	MgCl2 (125 mM)	DPBS
HA	4.5 μL	0 μL	0 μL	1.0 μL	119.5 μL
HA + IαI	4.5 μL	37.0 μL	0 μL	1.0 μL	82.5 μL
HA + TSG6	4.5 μL	0 μL	33.0 μL	1.0 μL	86.5 μL
HA + IαI + TSG6	4.5 μL	37.0 μL	33.0 μL	1.0 μL	49.5 μL

### Data analysis

To make comparisons between healthy and OA joints, data were tested for normality using a Shapiro-Wilk test. Right-skewed data, including both sandwich and competitive HA ELISA measurements, were transformed using a cube root transformation to achieve normality. Left-skewed synovial fluid viscosity data were log transformed to achieve normality. Following transformation, all normally distributed data were analyzed by comparing healthy and OA joints using an unpaired *t*-test and were reported as means ± SEM. Non-normally distributed data, including HA MW distributions (agarose gel electrophoresis and SS-nanopore), HC-HA immunoblotting, and synovial membrane HC-HA IF staining were reported as medians ± IQR and analyzed using Wilcoxon rank sum tests. For gene expression analysis, the median of all healthy samples was designated as 1.0, and fold change data were reported as medians ± ΙQR and analyzed using Wilcoxon rank sum tests. Synovial fluid chemokine data, including TNF-α, CCL2, CCL5, and CCL11, were compared using Wilcoxon rank sum tests. For the cytokines TNF-α and CCL5, samples with undetectable values of “0” were assigned an arbitrary value of “1” to enable statistical analysis between healthy and OA joints, with the arbitrary value being lower than the lowest detectable concentration. The majority of the samples had undetectable values for CCL3 and IL-1β; therefore, only descriptive statistics were reported for these chemokines. TSG-6-mediated HC-HA crosslinking viscosity data were analyzed using a one-way ANOVA, followed by Tukey-Kramer HSD post hoc tests. Significance was set at *α* < 0.05.

Correlation analyses of HA concentrations and HC-HA complex formation with synovial membrane gene expression data (*n* = 36) and synovial fluid cytokine concentrations (*n* = 74) were calculated using Spearman’s *ρ* correlation coefficients. All statistical analyses were performed using JMP Pro 13.1.0 software (SAS Institute Inc., Cary, NC). SF viscosity was plotted as a function of HA concentration by plotting the data as a second-order polynomial with the following constraints (equation: *y* = B0 + B1*x* + B2*x*^2^, constraint B0 = 1, B1 = 0) using Prism 7.04 (GraphPad Software, San Diego, CA). All graphs were generated using Prism. The average intra-assay coefficient of variability (CV) for HA ELISAs was generated using the individual CV value (percentage) from three plates and two replicates per sample.

## Results

### HA concentrations did not differ between healthy and OA joints

**Fig. 1:**
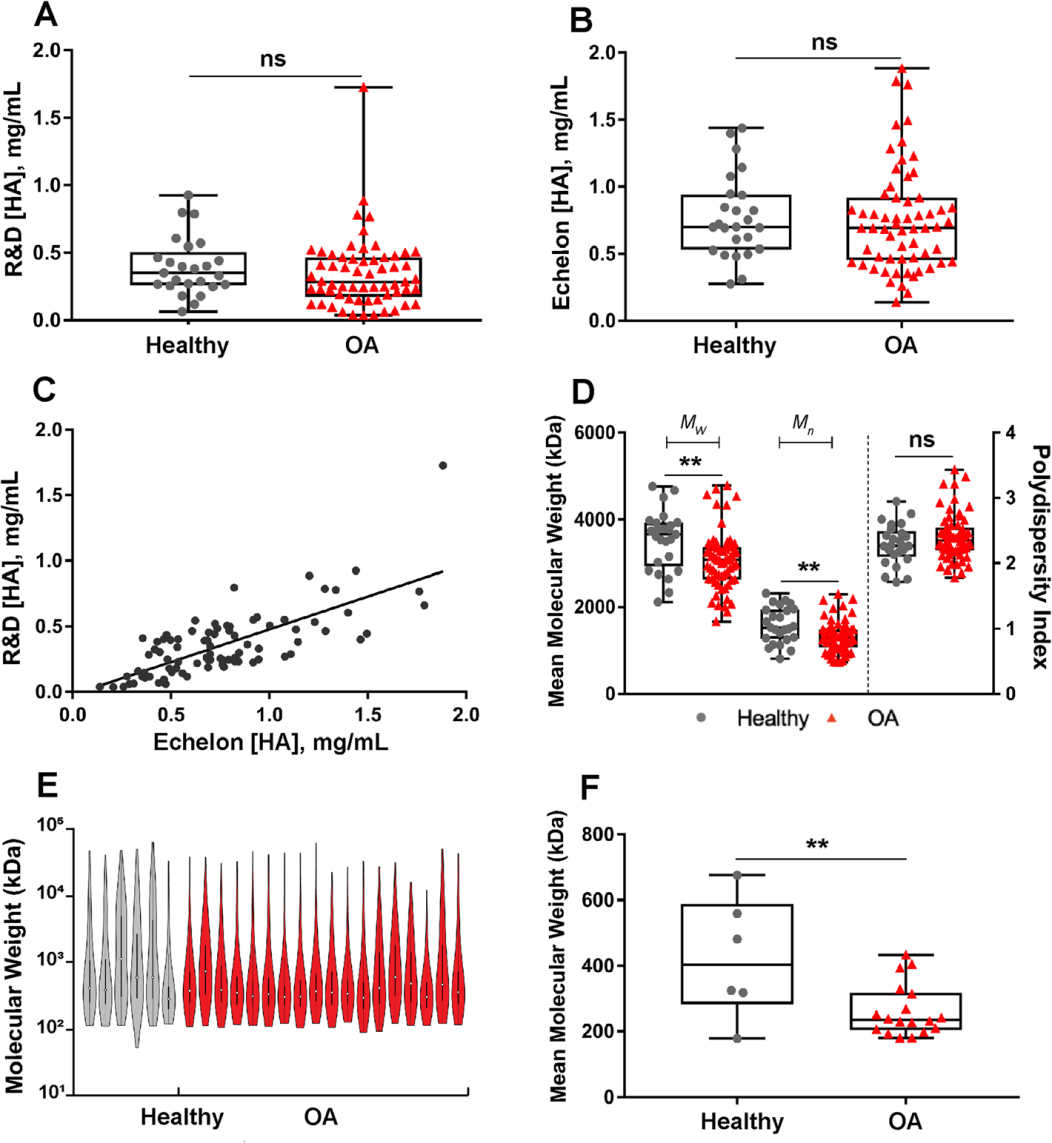
Hyaluronan (HA) molecular weight distribution differed between healthy and OA joints; HA concentration did not. HA concentrations were measured using **A** a sandwich ELISA and **B** a competitive ELISA (*n* = 25 healthy and 61 OA joints). **C** HA concentrations correlated between ELISAs (*R*^*2*^ = 0.5); however, the competitive ELISA yielded higher concentrations as compared to the sandwich ELISA. **D** HA molecular weights were measured by agarose gel electrophoresis and weight average molecular weight (*M*_*w*_), number average molecular weight (*M*_*n*_), and polydispersity index (*M*_*w*_/*M*_*n*_) were calculated for each sample. Both *M*_*w*_ and *M*_*n*_ were significantly decreased in OA versus healthy samples, while there was no significant difference in PDI between the two groups. **E** HA molecular weight distribution and **F** mean molecular weight of HA in a subset of OA (*n* = 18) and healthy (*n* = 6) samples measured by SS-nanopore. Mean molecular weight was significantly decreased in OA samples as compared to healthy samples. ns, not significant; *P* > 0.05. **P < 0.01. Data (**A**, **B**, **D**, **F**) are displayed as box-and-whisker plots representing the first and third quartiles, median, and spread of data for healthy and OA samples

The intra-assay CVs for the sandwich and competitive HA ELISAs were 6.5% and 8.1%, respectively. Synovial fluid HA concentrations demonstrated significant inter-individual variability in both healthy and OA groups (sandwich R&D ELISA range 0.04–1.73 mg/mL, competitive Echelon ELISA range 0.14–1.88 mg/mL). However, HA concentrations did not differ between healthy and OA joints for either the sandwich ELISA (median ± IQR 0.35 ± 0.3 and 0.29 ± 0.3 mg/mL, respectively) or the competitive ELISA (median ± IQR 0.70 ± 0.4 and 0.69 ± 0.5 mg/mL, respectively) (Fig. 1A, B). A strong correlation (Spearman’s *ρ* = 0.7, *P* < 0.0001) between both HA ELISAs was observed **(**Fig. 1C); however, the competitive ELISA reported greater synovial fluid HA concentrations, on average, as compared to the sandwich ELISA likely due to the known differential MW sensitivity of the methods.

### HA distributions were skewed to lower molecular weight variants in OA synovial fluid

HA distributions were first analyzed by agarose gel electrophoresis, and the data are shown as weight average (*M*_*w*_), number average (*M*_*n*_), and polydispersity index (PDI). As shown in Fig. 1D and Table 3, the HA distributions were skewed toward lower molecular weight variants in OA joints as compared to healthy joints as demonstrated by *M*_*w*_ (median ± IQR 3070 ± 740 and 3660 ± 1000, respectively, *P* = 0.002) and by *M*_*n*_ [median ± IQR 1260 ± 410 and 1520 ± 680, respectively, *P* = 0.003]. HA distributions were also measured by SS-nanopore for a subset of samples (*n* = 24), demonstrating the predominance of lower molecular weight variants in OA as compared to healthy joints as demonstrated by *M*_*w*_ (median ± IQR 3380 ± 1740 (OA) and 6450 ± 2100 (healthy), *P* = 0.005) and by *M*_*n*_ [median ± IQR 530 ± 300 (OA) and 1260 ± 1100 (healthy), *P* = 0.02] (Fig. 1E, F and Table 4). The differences in median values measured by the gel and nanopore approaches are likely a result of only a subset of specimens being analyzed by SS-nanopore. Other possible factors contributing to the differences could include the sensitivity limit of fluorescent imaging of the gels and a modest size bias in HA affinity bead capture.

**Table 3:** HA distributions measured by agarose gel electrophoresis

Measurement	Healthy (median ± IQR)	OA (median ± IQR)	***P*** value (healthy vs. OA)
Mw (kDa)	3660 ± 1000	3070 ± 740	0.002
Mn (kDa)	1520 ± 680	1260 ± 410	0.003
PDI	2.26 ± 0.4	2.38 ± 0.4	0.2
Number of samples	*n* = 25	*n* = 61	*n* = 86

**Table 4:** HA distributions measured by SS-nanopore

Measurement	Healthy (median ± IQR)	OA (median ± IQR)	***P*** value (healthy vs. OA)
Mw (kDa)	6450 ± 2100	3380 ± 1740	0.005
Mn (kDa)	1260 ± 1100	530 ± 300	0.02
PDI	4.97 ± 3.5	7.42 ± 4.2	0.4
Number of samples	*n* = 6	*n* = 18	*n* = 24

### Synovial fluid viscosity did not differ between healthy and OA joints, but did correlate with HA concentration in OA joints

**Fig. 2:**
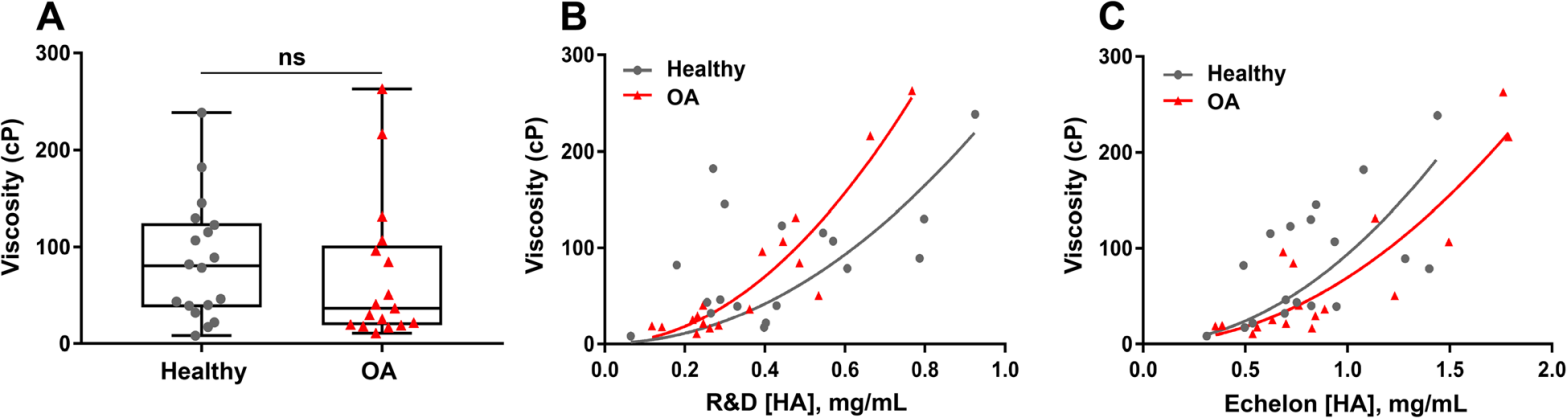
Synovial fluid viscosity and the correlation between viscosity and HA concentration. **A** Synovial fluid viscosity was measured by multiple particle-tracking microrheology (MPTM) and reported in centipoise (cP; *n* = 18 healthy, *n* = 17 OA). Data are displayed as box-and-whisker plots representing the first and third quartiles, median, and range of the viscosity values for healthy and OA samples. **B** A strong correlation between synovial fluid viscosity and HA concentration measured via a sandwich ELISA was observed in OA joints (*R*^2^ = 0.9, *P* < 0.0001, *n* = 17, equation: *y* = 1 + 434 *× x*^2^), but not in healthy joints (*R*^2^ = 0.1, *P* = 0.02, *n* = 18, equation: *y* = 1 + 256 *× x*^*2*^). **C** The synovial fluid viscosity was also found to have higher correlation with HA concentrations determined with competitive HA ELISA in OA joints (*R*^2^ = 0.8, *P* < 0.0001, *n* = 17, equation: *y* = 1 + 69 *× x*^2^) than in healthy joints (*R*^2^ = 0.2, *P* = 0.01, *n* = 18, equation: *y* = 1 + 93 *× x*^*2*^)

Similar to HA concentrations, synovial fluid viscosities demonstrated significant inter-individual variability in both healthy (range 8.45–238.75 cP) and OA groups (range 10.80–263.30 cP). Synovial fluid viscosity did not differ between healthy and OA joints, due to significant inter-individual variability (median ± IQR 80.6 ± 87.4 cP vs. 36.6 cP ± 82.4 cP, respectively) (Fig. 2A). A strong correlation between synovial fluid viscosity and HA concentration was observed in OA joints (*R*^2^ = 0.9, *P* < 0.0001, *n* = 17, equation: *y* = 1 + 434 *× x*^2^ and *R*^2^ = 0.8, *P* < 0.0001; *n* = 17, equation: *y* = 1 + 69 *× x*^*2*^) (Fig. 2B, C) but not in healthy joints (*R*^2^ = 0.1, *P* = 0.02, *n* = 18, equation: *y* = 1 + 256 *× x*^2^; *R*^2^ = 0.2, *P* = 0.01, *n* = 18, equation: *y* = 1 + 93 *× x*^2^) (Fig. 2B, C).

### TNF-α was increased and CCL11 was decreased in OA synovial fluid

**Fig. 3:**
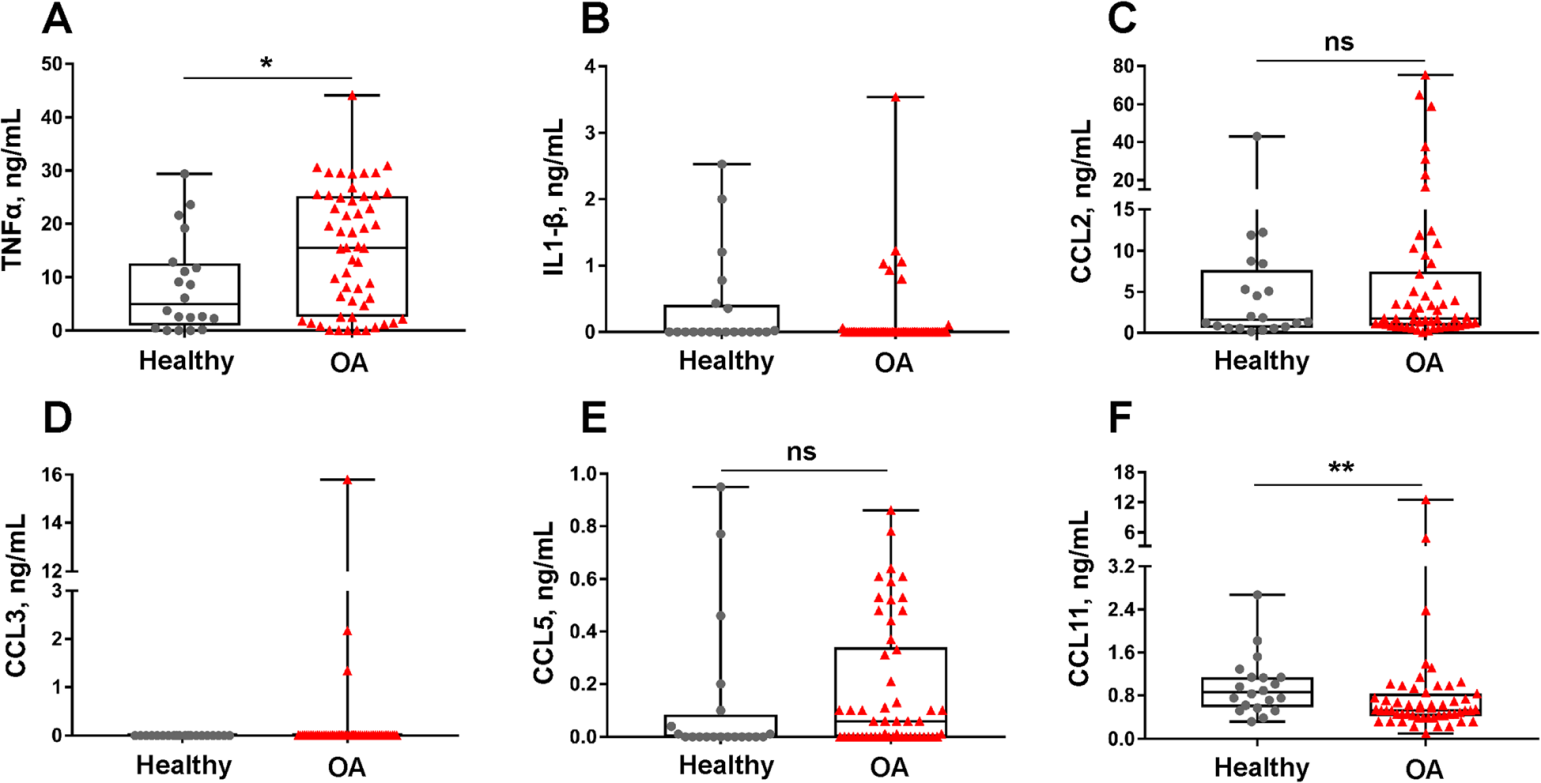
Synovial fluid concentrations of **A** TNF-α, **B** IL-1β, **C** CCL2, **D** CCL3, **E** CCL5, and **F** CCL11 were measured using a multiplex assay (*n* = 20 healthy and *n* = 54 OA joints). TNF-α was increased in OA joints, and CCL11 was decreased in OA joints. CCL2 and CCL5 did not differ between healthy and OA joints. ns, not significant; *P < 0.05, **P < 0.01. Data are displayed as box-and-whisker plots representing the first and third quartiles, median, and spread of concentrations for healthy and OA samples

TNF-α was significantly increased in OA (median ± IQR 15.56 ± 22.7 ng/mL) as compared to healthy joints (median ± IQR 4.97 ± 11.7 ng/mL, *P* = 0.03) (Fig. 3A, Table 5). There were no differences in CCL2 or CCL5 between healthy and OA joints (Fig. 3B–D, Table 5). The majority of synovial fluid samples demonstrated undetectable values for IL-1β and CCL3, with a median value of 0 for both healthy and OA joints; therefore, only descriptive statistics are reported (Fig. 3B, Table 5). Synovial fluid CCL11 was reduced in OA (median ± IQR 0.53 ± 0.4 ng/mL) as compared to healthy joints (median ± IQR 0.86 ± 0.6 ng/mL, *P* = 0.01) (Fig. 3F, Table 5).

**Table 5:** Cytokine and chemokine concentrations (ng/mL) in synovial fluid

Cytokine or chemokine	Healthy (median ± IQR)	OA (median ± IQR)	*P* values (healthy vs. OA)
TNF-α	4.97 ± 11.7	15.6 ± 22.7	0.03
IL-1β	0.00 ± 0.4	0.00 ± 0.0	NA
CCL2	1.61 ± 7.0	1.78 ± 6.6	0.59
CCL3	0.00 ± 0.0	0.00 ± 0.0	NA
CCL5	0.00 ± 0.1	0.06 ± 0.3	0.17
CCL11	0.86 ± 0.6	0.53 ± 0.4	0.01

NA: not analyzed

### *TSG6* gene expression was upregulated in OA synovial membrane and cartilage, and *HAS1* gene expression was downregulated in OA synovial membrane

**Fig. 4:**
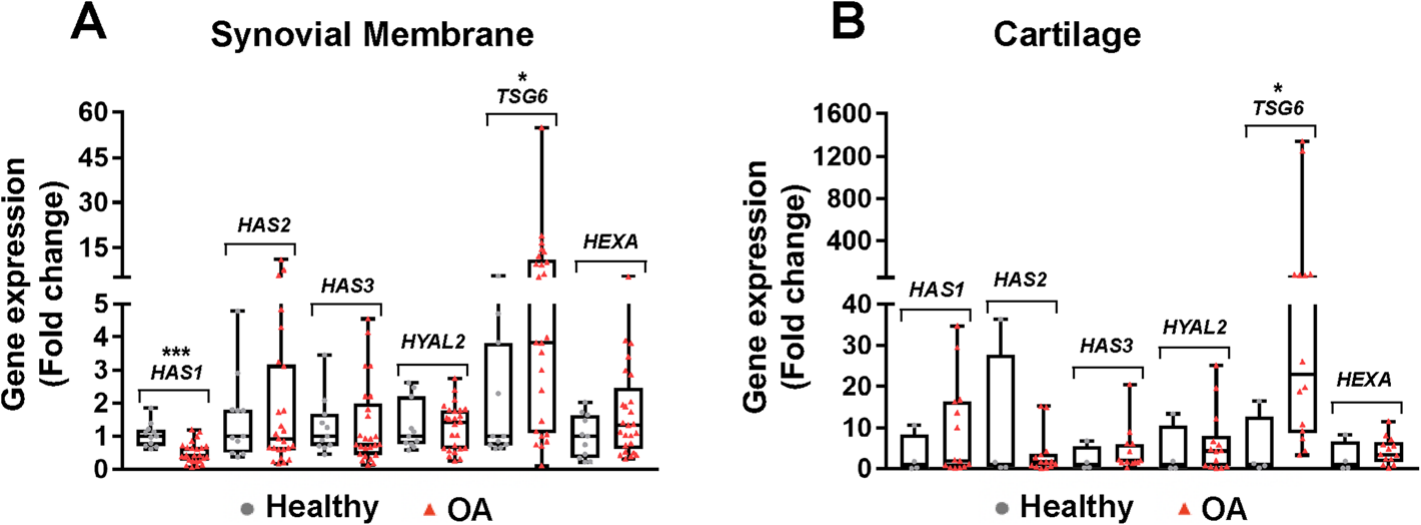
*TSG6* gene expression was increased in synovial membrane and cartilage from OA joints. Gene expression of hyaluronan synthases (*HAS1*, *HAS2*, *HAS3*), hyaluronidases and hexosaminadases (*HYAL2* and *HEXA*), and *TSG6* were detected by qRT-PCR (OA, osteoarthritis). Gene expression is reported as 2^−∆∆CT^ where ∆*C*_T_ = *C*_T_ (gene of interest) – *C*_T_ (18S rRNA), ∆∆*C*_T_ = (individual ∆C _T_) – healthy ∆*C*_T_ average and calculated as 2^−∆∆CT^. **A** Synovial membrane (*n* = 11 healthy and *n* = 25 OA) and **B** cartilage (*n* = 4 healthy and *n* = 14 OA) gene expression. *P < 0.05 , ****P < 0.0001. Data are displayed as box-and-whisker plots representing the first and third quartiles, median, and range of fold change gene expression levels for healthy and OA samples

Among 6 genes related to HA production and stability (*HAS1*, *HAS2*, *HAS3*, *TSG6*, *HYAL2*, and *HEXA*), only *TSG6* and *HAS1* differed between healthy and OA joints. *TSG6* gene expression was upregulated in both OA synovial membrane (median ± IQR 3.83 ± 9.9 (ΟΑ) vs. 1.00 ± 3.1 (healthy), *P* = 0.02, Fig. 4A) and cartilage (median ± IQR 22.87 ± 68.4 (OA) vs. 1.00 ± 12.4 (healthy), *P* = 0.02, Fig. 4B), whereas *HAS1* gene expression was downregulated in OA synovial membrane (median ± IQR = 0.42 ± 0.4 (OA) vs. 1.00 ± 0.5 (healthy), *P* = 0.0003, Fig. 4A).

### HC-HA complex formation was induced in OA and positively correlated with *TSG6* gene expression and synovial fluid TNF-α concentrations

**Fig. 5:**
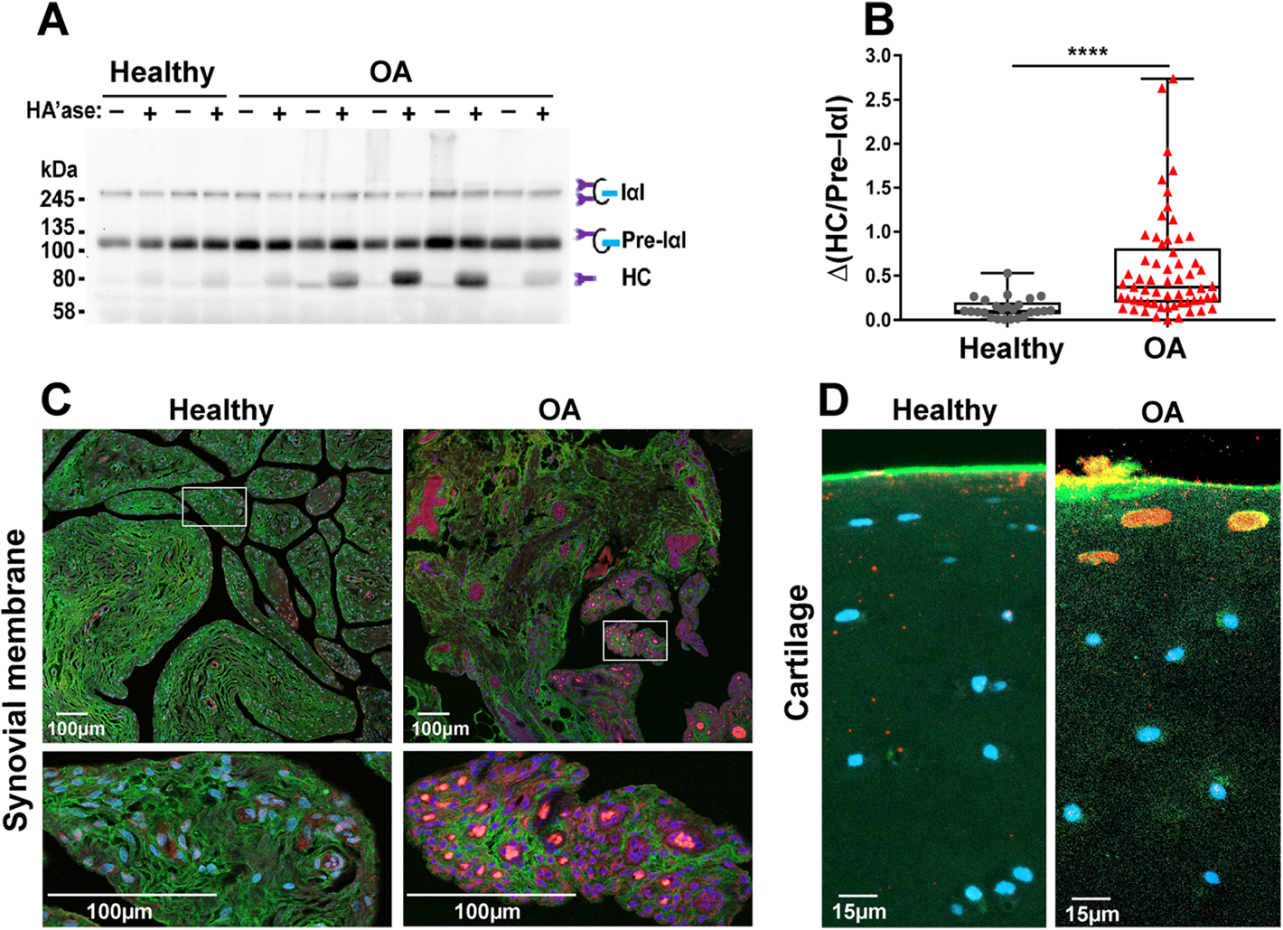
HC-HA complex formation was increased in OA joints. **A** Heavy chain-hyaluronic acid (HC-HA) complex formation was analyzed by western blot. Paired synovial fluid samples were run both with and without hyaluronidase (HAase) pre-digestion to release the HA-bound HC. Sample mixes (12 μL with the equivalent of 1 μL of undiluted SF) were loaded per lane. Lane “–” = endogenous HC; lane “+” = endogenous HC + HA-bound HC. **B** HC-HA relative absorbance unit (a.u.) = ∆(HC/Pre-IαI) = [HC (Lane “+”)/Pre-IαI]–[HC (Lane “–”)/Pre-IαI], used to compare healthy and OA joints (*n* = 25 healthy and *n* = 61 OA). ****P < 0.0001. Data are displayed as box-and-whisker plots representing the first and third quartiles, median, and spread of the HC-HA complex levels for healthy and OA samples. **C** The induction of HC-HA complex formation in synovial membrane tissues was evaluated by immunostaining. Paraffin-embedded sections were stained with HA binding protein (green), the Dako IαI antibody (red, to detect HC-HA complex), and DAPI (blue). Increased HC-HA was detected in the intimal and subintimal regions of OA synovial membrane tissue and was prominent within OA synovial tissue vasculature and lymphatics. **D** HC-HA was observed within chondrocyte lacunae of superficial zone chondrocytes in OA but not healthy articular cartilage

HC-HA complex formation was greater in OA as compared to healthy synovial fluid (median ± IQR 0.10 ± 0.1 vs. 0.38 ± 0.6, respectively, *P* < 0.0001, Fig. 5A, B**)**. HC-HA complex formation was positively correlated with synovial membrane *TSG6* gene expression (Spearman *ρ* = 0.4, *P* = 0.01, *n* = 36) and synovial fluid TNF-α concentrations (*ρ* = 0.3, *P* = 0.01, *n* = 74). CCL2 was also positively correlated with HC-HA complex formation (Spearman *ρ* = 0.3, *P* = 0.004, *n* = 74). HC-HA complexes were detected by an IαI antibody in both healthy and OA synovial membrane tissues; however, HC-HA staining was more prominent in OA as compared to healthy synovium **(**Fig. 5C). Synovial membrane HC-HA staining was more pronounced in OA (median ± IQR 2 ± 1.3, *n* = 6) than healthy joints (median ± IQR 1 ± 1, *n* = 5). HC-HA was detected in OA, but not healthy cartilage (Fig. 5D**)**. Within synovial membrane, HC-HA complex was identified in the intimal and subintimal regions and was especially prominent within OA vasculature and lymphatics (Fig. 5C). In cartilage, HC-HA was observed within superficial zone chondrocyte lacunae in OA tissues (Fig. 5D).

### TSG-6-mediated intermolecular crosslinking of HA

**Fig. 6:**
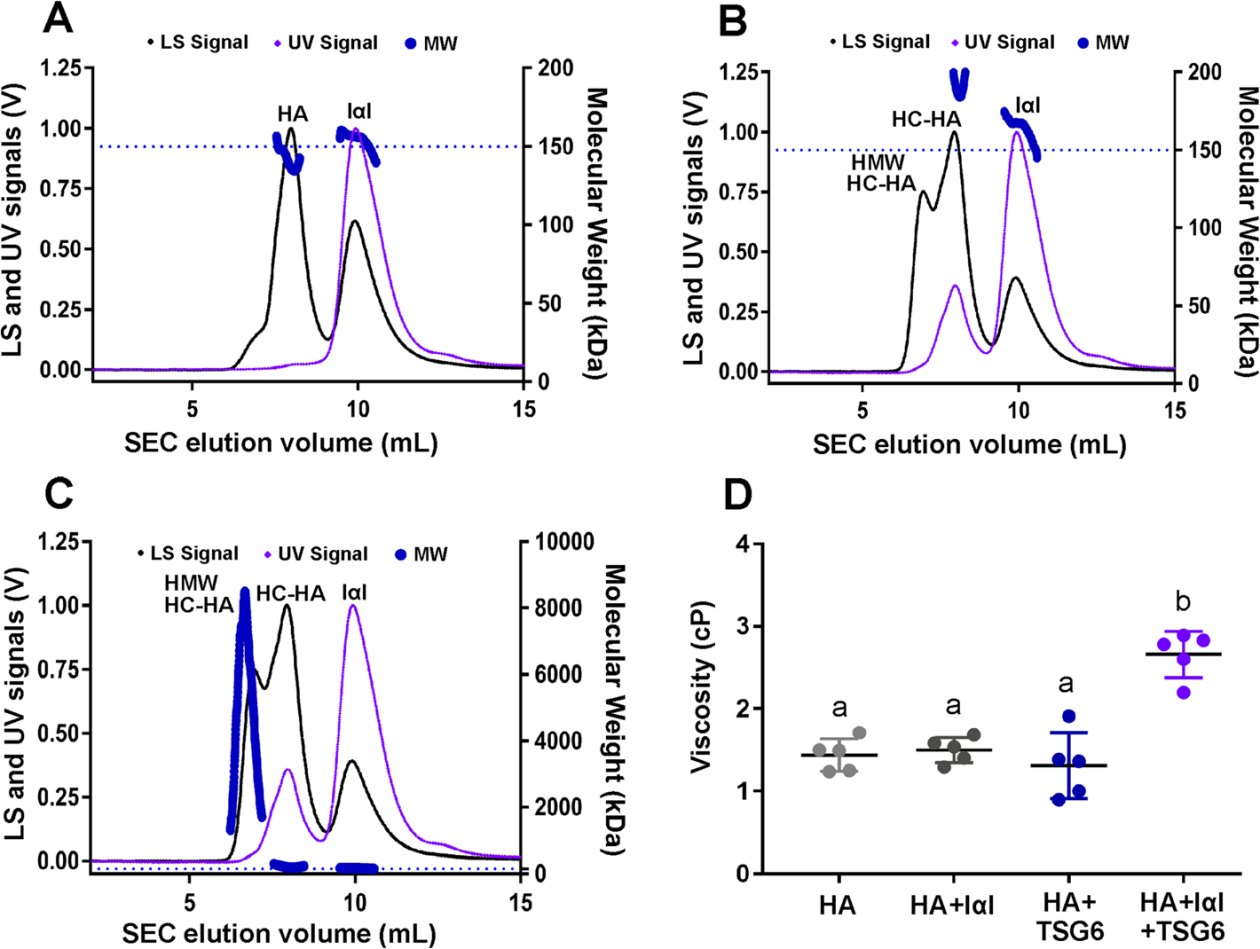
TSG6-mediated intermolecular crosslinking of HA. Size exclusion chromatography-multi-angle light scattering (SEC-MALS) was used to determine how the quaternary structure of monodisperse, 150 kDa HA was altered in the presence of TSG-6 and inter-alpha-inhibitor protein (IαIp), with one replicate (*n =* 1) for each treatment condition. **A** Control solution: HA and IαIp. Two significant peaks (HA and IαIp) were observed at their calculated MW of 141 kDa and 154 kDa, respectively. **B**, **C** HA, TSG-6, and IαIp. **C** was created by re-scaling the MW *y*-axis of **B** to show the high MW of HA aggregates. The light scattering (LS) shows 2 peaks with higher MW of ~ 220 kDa (equivalent to the MW of HC-HA complex, HC + HA = 75 kDa + 150 kDa) a weighted average of ~ 3288 kDa. The bold, dotted blue line indicates the calculated molecular weight across each peak, corresponding to MW (kDa) on the right *y*-axis. The dashed blue line represents the expected MW of 150 kDa for the monodisperse HA solution. **D** Viscosity increased in the TSG6-mediated HC-HA crosslinking reaction mixture (HA + IαI + TSG6) as compared to all other 3 controls. Data are displayed as scatter plots representing the mean ± SD (*n =* 5 technical replicates)

When HA was mixed only with IαIp without addition of TSG-6, two significant peaks were observed at approximately their calculated MW with HA 141 kDa and IαIp 154 kDa (in the range of 225 kDa IαI and 125 kDa Pre-IαI, unseparated by SEC-MALS) (Fig. 6A). As expected, the HA peak does not display a detectable UV signal under these conditions. When IαI was mixed only with TSG-6, a peak was observed for IαI at its calculated MW of 168 kDa. TSG-6 was not detectable due to the small amount used in the reaction (data not shown). When HA, TSG-6, and IαI were mixed and incubated together, in addition to the peak corresponding to the IαI MW, a peak with a calculated MW of 220 kDa was observed (Fig. 6B, C). This peak is proposed to represent a low MW HC-HA complex (designated as LMW HC-HA), as it agrees with the theoretical MW of the HC-HA complex (HC + HA = 75 kDa + 150 kDa = 225 kDa) and there is an increased UV signal, indicating co-migration with a protein element. A high MW peak was also observed in this condition, with calculated weights ranging from ~ 1 to 9 MDa and a weighted average of ~ 4.9 MDa (designated as HMW HC-HA), suggesting intermolecular crosslinking of HC-HA, resulting in large multi-molecular HA aggregates (Fig. 6B, C). The viscosity of TSG6-mediated HC-HA crosslinking product (HA + IαI + TSG6) was increased (median ± IQR 2.78 ± 0.5) as compared to the 3 controls (*P* < 0.0001) (Fig. 6D, Table 6). No differences in viscosity were observed between the 3 controls.

**Table 6:** Viscosities of TSG-6-mediated HC-HA crosslinking reaction mixtures

Reaction	Viscosity (cP) (median ± IQR)
HA	1.50 ± 0.4
HA + IαI	1.54 ± 0.3
HA + TSG6	1.37 ± 0.7
HA + IαI + TSG6	2.78 ± 0.5

## Discussion

In this naturally occurring equine OA model, synovial fluid HA concentration and viscosity did not differ between OA and healthy joints due to significant inter-individual variability; however, HA MW distributions were skewed to lower MW variants in OA joints. TNF-α SF concentrations, *TSG6* cartilage and synovial membrane mRNA expression, and HC-HA were all greater in OA joints as compared to healthy joints. HC-HA immunofluorescence staining was also much more prominent in OA as compared to healthy synovial membrane tissues and was localized to superficial zone chondrocytes in OA cartilage, but not healthy cartilage. For the in vitro experiments and in the presence of inter-alpha inhibitor (IαI), rhTSG6 resulted in a dramatic increase in the molecular size of 150-kDa monodisperse HA solution, presumably through intermolecular covalent crosslinking and HC-HA formation. To further investigate this *TSG6*-mediated phenomenon, we also demonstrated an increase in solution viscosity when a 50-kDa monodisperse HA solution was incubated with rhTSG6 and IαI. Taken together, these findings suggest that the TNF-α-TSG6-HC-HA signaling axis is active in equine PTOA and may result in relevant biophysical effects on synovial fluid and tissue HA.

Decreased SF HA concentrations and molecular weight distributions are often considered a hallmark of OA [12, 13, 41]. However, a prior study did not reveal differences in HA concentration between healthy and OA joints in an equine traumatic arthritis model [11], and a recent study did not detect a significant association between synovial fluid HA concentration and joint grade in human knee OA [10]. Here, two separate HA ELISAs, including both a sandwich and competitive inhibition ELISA, revealed no differences in HA concentration between healthy and OA joints. Of importance, the ELISAs did reveal significant variability in HA concentrations between individuals within both healthy and OA groups, suggesting that HA viscosupplementation may be more beneficial in some individuals than others. Both gel electrophoresis and SS-nanopore analyses revealed reduced HA size distributions as shown by both *M*_*w*_ and *M*_*n*_ in OA joints as compared to healthy joints, suggesting that changes in HA MW distribution may be more relevant than HA concentration changes alone. Previous studies have demonstrated a downward shift in SF HA MW in human OA and RA patients as compared to unaffected postmortem donors [41]. HA molecular weight distribution in SF has even been shown to be a successful predictor of OA progression; as HA MW distribution shifts downward, the odds of knee OA progression increases [13].

HA production in mammals is controlled by three isoforms of the enzyme hyaluronan synthase, *HAS1*, *HAS2*, and *HAS3* [42]. *HAS2* is critical to development and is found in greatest abundance in adult tissues [43], but any specific functions of each *HAS* isoform in various tissues are poorly understood [43]. In our study, *HAS2* and *HAS3* expression did not differ between OA and healthy joints, whereas *HAS1* expression was lower in OA as compared to healthy synovial membrane tissue. The function of *HAS1* is not entirely understood, but it may also regulate *HAS2* and metabolism of inflammatory matrices post-injury as *HAS1*-deficient mice develop chronic joint inflammation and severe intra-articular fibrosis following cartilage injury [44].

Consistent with the variability in SF HA concentrations, we did not detect significant differences in SF viscosity between healthy and OA joints. SF HA concentrations were strongly positively correlated with viscosity in OA joints, whereas correlations between HA and viscosity were weak for healthy joints. Our finding that SF viscosity alone is not a reliable marker for OA is corroborated by a recent study that determined that SF viscosity is not a suitable marker for human OA due to the large variance between individuals, and SF viscosity measurements commonly overlap between healthy and OA patients [45]. While there may be too much variation between individuals to use viscosity as a determinant for OA diagnosis, knowledge about SF viscosity may be useful for identifying rheologically or tribologically deficient synovial fluid phenotypes [46] or for identifying appropriate candidates for viscosupplementation. Our finding that HA concentrations correlated strongly with SF viscosity in OA patients is consistent with previous publications that identify HA as the primary determinant of SF viscosity [47, 48].

The TNF-α-TSG-6-HC-HA axis is active in naturally occurring equine carpal OA, suggesting that the horse could be a viable translational model to study the effects of HC-HA complex formation in PTOA. Increased expression of TNF-α has been documented in the synovium of human OA patients [49] and in some equine models [6, 50]. As TNF-α levels increase in inflammatory disease states, so too does the expression of genes stimulated by TNF-α, including TSG-6 [27]. Although we were unable to directly measure the TSG-6 protein in equine synovial fluid due to the unavailability of equine-specific antibodies or detection reagents, *TSG6* mRNA expression was greater in OA synovial membrane and cartilage tissues as compared to healthy tissues. To our knowledge, *TSG6* expression has not previously been evaluated in equine joint disease, but has been documented to increase following intra-articular administration of triamcinolone acetonide in exercised horses [51].

TSG-6 has been identified as a potential biomarker of OA progression [27, 29]; however, its role in osteoarthritis pathophysiology is less understood, and TSG-6 is thought to have both beneficial and adverse effects in the joint. TSG-6 is chondroprotective by blocking protease activation, including plasmin and MMPs [52], which mitigates ECM degradation in rodent PTOA and inflammatory arthritis models [26, 53]. In addition, TSG-6 transfers HC from IαI to HA, creating HC-HA complexes that may encourage cartilage matrix assembly [23]. However, IαI is unable to penetrate into intact intermediate and deep zones of cartilage, and increased TSG-6 activity may induce a deleterious state referred to as “futile synthesis” that may weaken cartilage [23]. HC-HA complex formation was increased in OA versus healthy synovial fluid, and HC-HA staining was much more prominent in OA cartilage and synovial membrane tissues, localizing to superficial zone chondrocytes and intimal, subintimal, and vascular regions of synovial membrane tissues. HC-HA can have a context-specific role in different tissues and disease states; while HC-HA crosslinking promotes inflammation in irritable bowel disease [21], it provides an anti-inflammatory matrix in wound healing [54]. In OA, HC-HA may play both beneficial and detrimental roles. While the HC-HA complex promotes leukocyte adhesion to HA matrices, it could accumulate and prevent the resolution of inflammation as in other tissues [18, 40, 55] or it may sequester inflammatory cells [56]. On the other hand, HC-HA may have the capability to encourage cartilage matrix assembly [23].

The crosslinked HC-HA complex plays important roles in several tissues. HA is necessary for the expansion of the cumulus cell-oocyte complex in female mammals and is a critical component of extracellular matrix organization [57]. HC is required to stabilize the cumulus extracellular matrix; because female *Tsg6* knockout mice fail to develop this matrix, they are sterile [58]. Covalent HC-HA complexes have also been discovered in the synovial fluid of human RA patients [59], yet the biophysical effect on these complexes on synovial fluid remained unclear [18]. Here, SEC-MALS analysis revealed a significant increase in HA MW upon the addition of TSG-6 and IαI, evidence for TSG-6-facilitated HA crosslinking. Similarly, microfluidic viscosity measurements also revealed an increase in the viscosity of HA solutions in the presence of TSG-6 and IαI, indicating that HA-HC crosslinking may be at least partially responsible for increased HA viscosity when TSG-6 is present.

As the samples used in this study were selected from equine patients with naturally occurring joint injury, there is inherent variability in disease severity and duration, similar to studies investigating PTOA in humans. This is both a strength and a limitation—the strength being that the findings more closely mimic clinical scenarios with increase translatability to humans; however, it can be more difficult to parse group and temporal effects. Advantages of the horse as an OA model is that this species naturally develops OA, the horse provides a source for a large quantity of SF, and advanced imaging and arthroscopy techniques are regularly used in the horse [60]. An additional limitation is that synovial fluid samples were only evaluated at a single time point; therefore, the relationship between TNF-α, TSG-6, and HC-HA formation and progression of OA could not specifically be evaluated in this study. It should also be noted that there is no current method to directly measure TSG-6 protein in the horse; while several anti-human antibodies were tested, none of these cross-reacted with equine TSG-6. In addition, while this study examines the biophysical effects of the TNF-α-TSG-6-HC-HA axis, the biological effects were not investigated here, including effects on synovial cell inflammatory cells.

To our knowledge, this study is the first to examine the biophysical effects of the TNF-α-TSG-6-HC-HA axis in synovial fluid in both healthy and OA samples and to reveal the distribution of HC-HA complexes in synovial and cartilage tissues. One notable result of this study is the use of SEC-MALs to confirm HA intermolecular aggregation as a function of TSG6-mediated crosslinking, with increased viscosity of these crosslinked products confirmed by microrheology viscosity measurements. At least two studies have identified a link between SF TSG-6 levels and OA progression in human subjects [29, 61], and others have demonstrated an increase in HC-HA complex formation in RA and in murine models of inflammatory arthritis [40, 59]. Our finding that SF HA concentration and viscosity varied significantly between and among healthy and OA individuals supports the hypothesis that OA is a heterogenous disease that contains multiple endotypes that may progress differently and respond differently to various treatments [62, 63].

The results of this study have several potential clinical applications. One such application is the use of synovial fluid TNF-α, TSG-6, and HC-HA as potential biomarkers of OA. All three molecules were greater in OA as compared to healthy joints and could therefore be useful diagnostic markers of disease state. While healthy and OA SF samples did not differ in HA concentration between groups, significant inter-individual variability in concentrations was present, as were reduced HA MW distributions in OA versus healthy joints. These results support the use of viscosity and HA MW distribution measurements in a clinical setting to differentiate disease endotypes and identify patients that may benefit from targeted viscosupplementation. Finally, this study motivates further investigation into the role of the TNF-α-TSG-6-HC-HA axis in joint disease, including how TSG-6 and HC-HA may affect infiltration, activation, and residence times of inflammatory cells and synovial fluid lubrication.

## Conclusions

Neither synovial fluid HA concentration nor viscosity was a reliable maker for naturally occurring equine OA due to substantial inter-individual variability; however, HA distributions were skewed to lower molecular weight variants in OA joints. The TNF-α-TSG-6-HC-HA axis is upregulated in equine OA, with evidence of HC-HA formation in several joint tissues, including synovial fluid, synovial membrane, and cartilage. TSG-6 induces macromolecular aggregation of HA, and the implications of HC-HA formation in multiple joint tissues, including synovial fluid, should be investigated in future studies.

## Supplementary Information

**Additional file 1.** Fig S1. Representative agarose gel electrophoretogram to measure HA distribution in equine synovial fluid. Healthy: Synovial fluid (SF) was collected from healthy horses (9 lanes); OA: SF samples were collected from horses with OA (26 lanes). HA MW markers are shown in the 1st lane.

**Additional file 2.** Table S1. Horse demographics and tissue disposition.

**Additional file 3.** Table S2. qRT-PCR checklist.

**Additional file 4.** Table S3. qRT-PCR cycle threshold (CT) values.

## Data Availability

The data that support the findings of this study are available on request from the corresponding author. The data are not publicly available due to privacy or ethical restrictions.

## Abbreviations

ACJ: Antebrachiocarpal joint
BSA: Bovine serum albumin
CCL: Chemokine ligand
COC: Cell-oocyte complex
HA: Hyaluronic acid
HABP: Hyaluronan binding protein
HAS: Hyaluronan synthase
HC-HA: Heavy chain-hyaluronic acid
HC: Heavy chains
HEXA: Hexosaminidase subunit α
HYAL: Hyaluronidase
IL-1β: Interleukin-1β
IαI: Inter-α-inhibitor
mAbs: Monoclonal antibodies
MCJ: Middle carpal joint
*M*_*n*_: Number average
MPTM: Multiple particle-tracking microrheology
MW: Molecular weight
*M*_*w*_: Weight average
OA: Osteoarthritis
PBS: Phosphate-buffered saline
PDI: Polydispersity index
PTOA: Post-traumatic osteoarthritis
qRT-PCR: Quantitative real-time polymerase chain reaction
SEC-MALS: Size exclusion chromatography with multi-angle light scattering
SF: Synovial fluid
SM: Synovial membrane
SS: Solid state
TAE: Tris-acetate-EDTA
TSG-6: TNF-α-stimulated gene 6
