# Supplementary material for: Hyaluronic acid synthesis, degradation, and crosslinking in equine osteoarthritis: TNF-α-TSG-6-mediated HC-HA formation
Source: Arthritis Res Ther. 2021 Aug 20;23:218. doi: 10.1186/s13075-021-02588-7 (PMC8377964; doi:10.1186/s13075-021-02588-7)
Supplement: Supplementary file 1 — Additional file 1. Fig S1. Representative agarose gel electrophoretogram to measure HA distribution in equine synovial fluid. Healthy: Synovial fluid (SF) was collected from healthy horses (9 lanes); OA: SF samples were collected from horses with OA (26 lanes). HA MW markers are shown in the 1st lane. [file 13075_2021_2588_MOESM1_ESM.pdf]

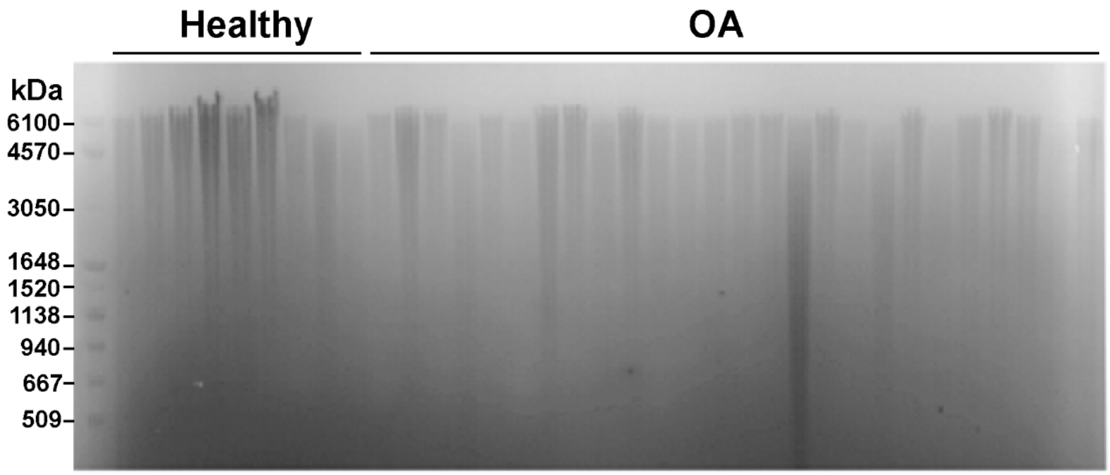

Fig S1. Representative agarose gel electrophoretogram to measure HA distribution in equine synovial fluid. Healthy: Synovial fluid (SF) was collected from healthy horses (9 lanes); OA: SF samples were collected from horses with OA (26 lanes). HA MW markers are shown in the 1st lane.
