# Supplementary material for: Hyaluronic acid synthesis, degradation, and crosslinking in equine osteoarthritis: TNF-α-TSG-6-mediated HC-HA formation
Source: Arthritis Res Ther. 2021 Aug 20;23:218. doi: 10.1186/s13075-021-02588-7 (PMC8377964; doi:10.1186/s13075-021-02588-7)
Supplement: Supplementary file 2 — Additional file 2. Table S1. Horse demographics and tissue disposition. [file 13075_2021_2588_MOESM2_ESM.pdf]

Table S1. Horse demographics and tissue disposition

Horse number	Breed	Age (years)	Sex	OA grade	Healthy vs OA	SF volume (mL)	R&D HA ELISA	Echelon HA ELISA	HA gel	HC-HA complex	Cytokine	qRT-PCR		Immunostaining		MPTM	SS-nanopore
												SM	Cartilage	SM	Cartilage		
H1	TB	3	Castrated male	0	Healthy	9	x	x	x	x	x					x	x
H2	QH	3	Castrated male	0	Healthy	6.5	x	x	x	x	x					x	
H3	WB	3	Female	0	Healthy	NA	x	x	x	x	x	x	x			x	
H4	Mixed	5	Castrated male	0	Healthy	3	x	x	x	x	x	x	x	x	x	x	x
H5	TB	9	Female	0	Healthy	7	x	x	x	x	x					x	
H6	TB	4	Female	0	Healthy	6	x	x	x	x	x	x		x		x	x
H7	TB	7	Castrated male	0	Healthy	NA	x	x	x	x		x					
H8	TB	7	Female	0	Healthy	NA	x	x	x	x		x					
H9	TB	5	Female	0	Healthy	NA	x	x	x	x	x	x	x			x	x
H10	STB	3	Castrated male	0	Healthy	5.5	x	x	x	x	x					x	
H11	TB	6	Female	0	Healthy	NA	x	x	x	x		x	x			x	
H12	STB	5	Castrated male	0	Healthy	7.5	x	x	x	x	x						x
H13	TB	3	Female	0	Healthy	6.5	x	x	x	x	x					x	
H14	Cross	5	Female	0	Healthy	5	x	x	x	x	x					x	
H15	Cross	5	Female	0	Healthy	5	x	x	x	x	x					x	
H16	STB	3	Castrated male	0	Healthy	5.8	x	x	x	x	x						
H17	Paint	2	Intact male	0	Healthy	5.5	x	x	x	x	x	x		x	x	x	x
H18	TB	4	Female	0	Healthy	3.5	x	x	x	x	x	x		x	x	x	
H19	TB	6	Intact male	0	Healthy	NA	x	x	x	x		x					
H20	TB	10	Castrated male	0	Healthy	5.5	x	x	x	x	x					x	
H21	TB	11	Female	0	Healthy	5.5	x	x	x	x	x						
H22	Cross	5	Female	0	Healthy	5	x	x	x	x	x					x	
H23	TB	5	Intact male	0	Healthy	NA	x	x	x	x		x					
H24	Cross	5	Female	0	Healthy	4	x	x	x	x	x					x	
H25	TB	5	Female	0	Healthy	9	x	x	x	x	x					x	
OA1	TB	22	Female	1	OA	7	x	x	x	x	x						
OA2	TB	1	Female	1	OA	1	x	x	x	x	x						
OA3	TB	2	Female	1	OA	5.5	x	x	x	x	x						
OA4	TB	2	Female	1	OA	4	x	x	x	x	x						

Horse number	Breed	Age (years)	Sex	OA grade	Healthy vs OA	SF volume (mL)	R&D HA ELISA	Echelon HA ELISA	HA gel	HC-HA complex	Cytokine	qRT-PCR		Immunostaining		MPTM	SS-nanopore
												SM	Cartilage	SM	Cartilage		
OA5	TB	2	Female	1	OA	4.5	x	x	x	x	x						
OA6	TB	2	Intact male	1	OA	7.5	x	x	x	x	x	x	x	x	x	x	x
OA7	TB	3	Intact male	1	OA	6	x	x	x	x	x	x					
OA8	TB	4	Female	1	OA	NA	x	x	x	x	x	x	x				
OA9	STB	5	Castrated male	1	OA	7	x	x	x	x	x						
OA10	TB	2	Intact male	1	OA	NA	x	x	x	x	x	x				x	x
OA11	TB	9	Female	1	OA	4.5	x	x	x	x	x						
OA12	TB	4	Female	1	OA	1.8	x	x	x	x	x	x					
OA13	TB	3	Female	1	OA	7	x	x	x	x	x	x				x	x
OA14	TB	2	Female	1	OA	4	x	x	x	x	x						
OA15	STB	3	Intact male	1	OA	2.8	x	x	x	x	x	x				x	x
OA16	TB	3	Female	1	OA	1.2	x	x	x	x	x						
OA17	TB	2	Female	1	OA	9.5	x	x	x	x	x						
OA18	TB	4	Female	1	OA	NA	x	x	x	x	x						
OA19	STB	2	Female	1	OA	5	x	x	x	x	x		x				
OA20	TB	2	Castrated male	1	OA	3.6	x	x	x	x	x						
OA21	STB	2	Castrated male	1	OA	10	x	x	x	x	x	x				x	x
OA22	TB	5	Intact male	1	OA	7											x
OA23	TB	3	Female	2	OA	10	x	x	x	x	x						
OA24	TB	4	Castrated male	2	OA	0.5	x	x	x	x	x						
OA25	STB	4	Female	2	OA	NA	x	x	x	x		x					
OA26	TB	2	Intact male	2	OA	8	x	x	x	x	x						
OA27	TB	3	Female	2	OA	5.5	x	x	x	x	x						
OA28	TB	2	Female	2	OA	10	x	x	x	x	x	x	x			x	x
OA29	TB	4	Female	2	OA	1.2	x	x	x	x	x				x		
OA30	TB	4	Female	2	OA	1.2	x	x	x	x	x	x				x	x
OA31	QH	13	Female	2	OA	2	x	x	x	x	x						
OA32	TB	2	Female	2	OA	1.5	x	x	x	x	x	x	x		x	x	x
OA33	TB	5	Female	2	OA	5	x	x	x	x	x						
OA34	TB	3	Castrated male	2	OA	4	x	x	x	x	x	x				x	x
OA35	TB	3	Female	2	OA	5	x	x	x	x	x		x			x	x
OA36	TB	3	Female	2	OA	3.5	x	x	x	x	x			x			

Horse number	Breed	Age (years)	Sex	OA grade	Healthy vs OA	SF volume (mL)	R&D HA ELISA	Echelon HA ELISA	HA gel	HC-HA complex	Cytokine	qRT-PCR		Immunostaining		MPTM	SS-nanopore
												SM	Cartilage	SM	Cartilage		
OA37	TB	3	Intact male	2	OA	3	x	x	x	x	x						
OA38	STB	5	Intact male	2	OA	5	x	x	x	x	x						
OA39	TB	4	Female	2	OA	4	x	x	x	x	x	x	x			x	x
OA40	TB	4	Female	2	OA	NA	x	x	x	x		x					
OA41	TB	3	Female	2	OA	1	x	x	x	x	x	x		x		x	
OA42	TB	4	Intact male	2	OA	4.5	x	x	x	x	x						
OA43	TB	4	Female	2	OA	1.4	x	x	x	x	x						
OA44	TB	2	Female	2	OA	NA	x	x	x	x		x	x				
OA45	TB	3	Female	2	OA	5	x	x	x	x	x						
OA46	TB	2	Female	2	OA	2.5	x	x	x	x	x		x				
OA47	STB	7	Castrated male	2	OA	NA	x	x	x	x		x	x				
OA48	QH	13	Castrated male	2	OA	3	x	x	x	x	x						
OA49	STB	3	Castrated male	3	OA	5.1	x	x	x	x	x						
OA50	QH	7	Female	3	OA	3	x	x	x	x	x	x				x	
OA51	QH	10	Female	3	OA	3	x	x	x	x	x						x
OA52	TB	4	Female	3	OA	NA	x	x	x	x		x					
OA53	TB	6	Castrated male	3	OA	3	x	x	x	x	x						
OA54	QH	20	Castrated male	3	OA	0.5	x	x	x	x		x			x		
OA55	TB	3	Castrated male	3	OA	5	x	x	x	x	x						x
OA56	TB	5	Female	3	OA	NA	x	x	x	x	x	x	x			x	x
OA57	TB	2	Castrated male	3	OA	8.5	x	x	x	x	x						
OA58	STB	3	Intact male	3	OA	NA	x	x	x	x		x	x				
OA59	TB	6	Female	3	OA	7.5	x	x	x	x	x		x	x	x		
OA60	QH	9	Castrated male	3	OA	6.5	x	x	x	x	x	x				x	x
OA61	TB	4	Female	3	OA	NA	x	x	x	x	x	x	x			x	x
OA62	TB	19	Female	3	OA	5	x	x	x	x	x					x	x
OA63	TB	7	Castrated male	3	OA	5.5								x			
						Mean = 4.9											

NA: Not applicable, this information was not available from patient records.

QH: Quarter Horse
STB: Standardbred
TB: Thoroughbred
WB: Warmblood
