# Supplementary material for: Hyaluronic acid synthesis, degradation, and crosslinking in equine osteoarthritis: TNF-α-TSG-6-mediated HC-HA formation
Source: Arthritis Res Ther. 2021 Aug 20;23:218. doi: 10.1186/s13075-021-02588-7 (PMC8377964; doi:10.1186/s13075-021-02588-7)
Supplement: Supplementary file 3 — Additional file 3. Table S2. qRT-PCR checklist. [file 13075_2021_2588_MOESM3_ESM.pdf]

Table S2. qRT-PCR checklist

Sample preservation/RNA template	Details
Source	Synovial membrane and articular cartilage tissues were collected from the carpal joints of horses (*Equus caballus*) with healthy joints or osteoarthritic (OA) joints.
Sample preservation	Synovial membrane and articular cartilage tissues were frozen in liquid nitrogen after collection and stored at -70°C.
Sample grinding and storage	Frozen samples were crushed and ground into fine powder with a mortar & pestle and stored at -70°C.
RNA extraction and removal of genomic DNA	Total RNA was extracted from synovial membrane using the E.Z.N.A Tissue RNA Kit (Omega BioTek, Inc., Norcross, GA) or from cartilage with the RNeasy Lipid Tissue Mini Kit (QIAGEN Sciences Inc., Germantown, MD). Residual genomic DNA was removed by DNase I on-column digestion. The extracted total RNA was stored at -70°C.
RNA concentration and integrity	RNA concentration and quality were assessed using a Tecan Spark 10M multimode plate reader with a NanoQuant Plate™ (to detect 16 samples per assay).
**Genes assayed**	**Accession number**
Equine *HAS1*	XM_023650323.1
Equine *HAS2*	NM_001081801.2
Equine *HAS3*	XM_023637194.1
Equine *TSG6*	NM_001081906.1
Equine *HYAL2*	XM_014731656.1
Equine *HEXA*	XM_001494311.4
Equine 18S rRNA	NR_046271.1
**Primers and amplicon**	**Sequences or size**
EqHAS1 Forward	GCGATACTGGGTGGCCTTCAATGT
EqHAS1 Reverse	CTGTATAGGCCTAGGGGACCACTG
EqHAS1 amplicon size	90-bp
EqHAS2 Forward	GGCCGGTCGTCTCAAATTCA
EqHAS2 Reverse	TCACAATGCATCTTGTTCAGCTC
EqHAS2 amplicon size	132-bp
EqHAS3 Forward	CGTGGGCGCATCTGGAACATT
EqHAS3 Reverse	CTCTGCATTGCCCCGAAGGAAG
EqHAS3 amplicon size	99-bp
EqTSG6 Forward	ATCCTGAGCAGCCCCTAACA
EqTSG6 Reverse	TTGAATCCCCATCCGTGAGC
EqTSG6 amplicon size	108-bp
EqHYAL2 Forward	CTCACAGGGCTTAGCGAGAT
EqHYAL2 Reverse	GGTACTGGCAGGTCTCCGTG
EqHYAL2 amplicon size	124-bp
EqHEXA Forward	AAGGAGCTGGAACTGGTCAC
EqHEXA Reverse	TCAGGGGTACCGTCAAATGC
EqHEXA amplicon size	137-bp
Eq18S rRNA Forward	GGCGTCCCCCAACTTCTT
Eq18S rRNA Reverse	AGGGCATCACAGACCTGTTATTG
Eq18S rRNA amplicon size	77-bp
Primer design	Primers were designed using NCBI Primer 3-Blast or Lasergene (DNASTAR, Madison, WI).
**SYBR Green qRT-PCR Protocol**	**Details**
SYBR Green qRT-PCR kit	SYBR Green RNA-to-C_T one-step kit (Applied Biosystems, Foster City, CA) was used.

RT & qPCR	RT and qPCR were carried out in the same well using the SYBR Green one-step kit. Equal amounts of total RNA (30 ng per reaction in 20 µl reaction mix for synovial membrane or 15 ng in 10 µl reaction mix for cartilage) were used, and all samples were run in duplicate.
No template control (NTC)	NTCs were included in each run with each primer set.
Calculation of relative gene expression	Relative gene expression was analyzed using the $2^{-\Delta\Delta CT}$ method. The fold change was calculated using the median of healthy samples as 1.
Data Normalization	All data were normalized to equine 18S rRNA values.
