# Supplementary material for: Hyaluronic acid synthesis, degradation, and crosslinking in equine osteoarthritis: TNF-α-TSG-6-mediated HC-HA formation
Source: Arthritis Res Ther. 2021 Aug 20;23:218. doi: 10.1186/s13075-021-02588-7 (PMC8377964; doi:10.1186/s13075-021-02588-7)
Supplement: Supplementary file 4 — Additional file 4. Table S3. qRT-PCR cycle threshold (CT) values. [file 13075_2021_2588_MOESM4_ESM.pdf]

Table S3. qRT-PCR cycle threshold (CT) values

Synovial membrane

Sample No.	Healthy vs OA	HAS1 CT (duplicate)	HAS2 CT (duplicate)	HAS3 CT (duplicate)	HYAL2 CT (duplicate)	TSG6 CT (duplicate)	HexA CT (duplicate)	18S CT (duplicate)	HAS1 CT Avg.	HAS2 CT Avg.	HAS3 CT Avg.	HYAL2 CT Avg.	TSG6 CT Avg.	HexA CT Avg.	18S CT Avg.
H4	Healthy	26.6	27.4	27.7	27.2	25.0	26.0	13.0	26.6	27.3	27.7	27.3	25.0	26.0	13.0
		26.6	27.3	27.6	27.4	25.0	25.9	13.0							
H6	Healthy	26.1	29.5	29.3	28.2	27.2	27.4	13.0	26.1	29.5	29.3	28.2	27.2	27.4	13.0
		26.1	29.6	29.3	28.3	27.2	27.4	13.0							
H7	Healthy	26.2	28.4	28.6	28.7	27.2	28.4	12.8	26.2	28.3	28.5	28.5	27.2	28.4	12.8
		26.1	28.2	28.5	28.4	27.1	28.3	12.7							
H8	Healthy	26.6	29.6	28.1	28.2	27.4	28.0	12.9	26.6	29.5	28.1	28.0	27.4	28.0	12.9
		26.6	29.4	28.2	27.8	27.5	28.0	12.8							
H9	Healthy	25.5	27.6	27.9	28.6	26.8	26.3	12.9	25.5	27.6	27.9	28.6	26.9	26.3	12.9
		25.5	27.6	28.0	28.6	26.9	26.4	12.9							
H11	Healthy	26.1	28.6	29.3	29.1	27.0	27.0	13.0	26.1	28.7	29.4	29.1	27.0	27.1	13.0
		26.1	28.7	29.5	29.1	27.0	27.2	13.1							
H13	Healthy	25.8	26.2	26.9	28.4	24.5	26.9	13.0	25.9	26.2	26.9	28.4	24.5	26.9	12.9
		25.9	26.2	26.9	28.4	24.4	26.9	12.9							
H17	Healthy	25.8	27.7	29.2	27.2	24.8	26.3	13.0	25.8	27.8	29.3	27.2	24.8	26.3	13.1
		25.8	27.8	29.3	27.2	24.8	26.2	13.1							
H18	Healthy	25.8	26.9	28.5	27.0	25.8	26.2	13.0	25.8	27.0	28.4	27.0	25.8	26.3	13.0
		25.8	27.0	28.3	27.1	25.7	26.3	13.0							
H19	Healthy	26.4	29.9	28.9	29.2	27.5	29.0	12.9	26.5	29.8	28.9	29.1	27.5	29.0	12.9
		26.5	29.7	28.9	29.1	27.5	29.0	12.9							
H23	Healthy	25.0	28.8	29.8	28.7	27.4	29.0	12.8	25.0	28.6	29.8	28.7	27.3	29.0	12.9
		25.1	28.5	29.9	28.7	27.2	29.1	12.9							
OA6	OA	26.4	29.1	27.0	28.2	25.3	26.1	12.9	26.4	29.2	27.0	28.3	25.3	26.1	12.9
		26.5	29.3	27.1	28.4	25.3	26.2	12.9							
OA7	OA	27.6	29.1	30.5	28.3	23.5	27.2	13.1	27.7	29.2	30.5	28.2	23.5	27.2	13.4
		27.7	29.2	30.5	28.1	23.6	27.3	13.7							
OA8	OA	25.8	26.4	30.0	28.8	27.7	28.0	12.9	25.8	26.3	29.9	28.8	27.4	27.9	12.9
		25.8	26.2	29.7	28.8	27.1	27.9	12.8							
OA10	OA	28.3	30.9	27.5	30.2	27.2	28.7	13.4	28.4	31.0	27.5	30.2	27.3	28.6	13.3
		28.5	31.0	27.5	30.1	27.4	28.5	13.3							
OA12	OA	27.8	29.0	29.7	30.6	27.5	28.8	13.5	27.8	29.0	29.7	30.6	27.6	28.8	13.4

		27.9	29.1	29.7	30.6	27.7	28.8	13.2							
OA13	OA	26.0	26.9	28.6	27.1	25.0	25.4	12.9	26.0	26.8	28.7	27.1	25.0	25.4	12.9
		26.0	26.8	28.8	27.1	25.0	25.4	12.9							
OA15	OA	28.0	30.4	28.9	29.0	26.7	27.8	13.0	28.0	30.3	28.8	29.2	26.7	27.9	12.9
		28.0	30.2	28.7	29.4	26.8	28.0	12.9							
OA21	OA	29.2	31.3	30.3	28.9	27.2	25.8	12.9	29.2	31.0	30.4	29.0	27.3	25.8	12.9
		29.3	30.7	30.5	29.1	27.3	25.9	12.8							
OA25	OA	27.6	25.8	27.9	27.7	23.5	24.9	13.3	27.6	25.9	27.9	27.6	23.5	24.9	13.3
		27.6	26.0	27.9	27.6	23.5	24.9	13.2							
OA28	OA	29.7	31.2	32.1	30.7	30.4	28.3	13.3	29.9	31.2	32.0	30.7	30.4	28.6	13.3
		30.1	31.2	31.9	30.8	30.4	28.8	13.3							
OA30	OA	26.8	29.1	27.9	29.1	25.7	27.3	13.0	26.8	29.2	27.9	29.1	25.7	27.3	13.0
		26.8	29.2	27.9	29.0	25.8	27.4	12.9							
OA32	OA	29.1	29.5	30.1	27.2	24.0	25.8	13.2	29.2	29.4	30.2	27.2	24.0	25.7	13.2
		29.3	29.4	30.2	27.2	24.0	25.7	13.2							
OA34	OA	26.4	30.0	26.7	27.6	23.6	26.6	13.0	26.5	30.0	26.7	27.5	23.6	26.5	12.9
		26.6	30.1	26.7	27.5	23.6	26.5	12.9							
OA39	OA	30.3	28.4	31.5	29.2	26.5	26.8	13.0	30.1	28.5	31.3	29.1	26.4	26.9	13.0
		29.9	28.5	31.2	29.1	26.4	27.0	13.0							
OA40	OA	28.0	29.8	29.4	27.8	21.6	26.3	13.3	28.0	29.6	29.5	27.8	21.5	26.4	13.3
		28.0	29.4	29.6	27.9	21.5	26.4	13.3							
OA41	OA	26.7	27.8	28.0	27.6	25.1	26.0	12.8	26.7	27.7	28.1	27.6	25.1	26.1	13.0
		26.6	27.6	28.1	27.6	25.2	26.2	13.1							
OA44	OA	26.5	25.9	29.2	27.7	24.2	26.9	12.8	26.5	25.8	29.0	27.6	24.2	26.8	12.8
		26.4	25.7	28.8	27.5	24.1	26.7	12.8							
OA47	OA	27.0	28.5	31.0	27.8	23.2	25.4	13.2	26.9	28.4	30.9	27.9	23.2	25.5	13.2
		26.9	28.2	30.9	27.9	23.2	25.5	13.2							
OA50	OA	27.4	28.3	29.7	27.9	25.0	25.0	13.0	27.4	28.3	29.4	27.8	25.0	25.1	13.0
		27.4	28.3	29.2	27.8	25.1	25.1	13.0							
OA52	OA	28.0	29.1	26.5	29.4	27.0	28.8	13.0	27.4	29.0	26.5	29.3	26.8	28.6	12.9
		26.9	29.0	26.5	29.3	26.7	28.4	12.9							
OA54	OA	25.6	24.9	27.4	27.9	23.3	26.7	12.8	25.6	24.9	27.5	27.8	23.2	26.4	12.8
		25.7	25.0	27.6	27.7	23.2	26.0	12.8							
OA56	OA	26.2	26.6	28.6	27.4	22.6	26.8	12.8	26.1	26.6	28.6	27.4	22.5	26.8	12.8
		26.0	26.7	28.6	27.4	22.5	26.8	12.8							

OA58	OA	27.4	27.9	29.5	28.0	23.9	25.1	13.1	27.4	27.9	29.5	28.0	23.9	25.2	13.1
		27.4	27.9	29.4	28.1	23.9	25.2	13.1							
OA60	OA	28.1	29.2	29.2	28.6	24.9	26.8	13.5	28.1	29.3	29.1	28.6	24.9	26.8	13.2
		28.1	29.3	29.1	28.7	24.9	26.8	13.0							
OA61	OA	27.9	26.3	29.2	27.8	24.9	27.5	12.8	27.9	26.1	29.1	27.7	24.8	27.3	12.8
		27.8	25.9	28.9	27.5	24.8	27.1	12.8							

Cartilage

Sample No.	Healthy vs OA	HAS1 CT (duplicate)	HAS2 CT (duplicate)	HAS3 CT (duplicate)	HYAL2 CT (duplicate)	TSG6 CT (duplicate)	HexA CT (duplicate)	18S CT (duplicate)	HAS1 CT Avg.	HAS2 CT Avg.	HAS3 CT Avg.	TSG6 CT Avg.	HYAL2 CT Avg.	HexA CT Avg.	18S CT Avg.
H3	Healthy	36.2	32.2	35.6	34.9	36.1	32.4	13.9	37.9	32.4	34.9	36.1	34.4	32.6	13.9
		39.7	32.6	34.2	34.0	36.1	32.8	13.9							
H4	Healthy	35.2	32.4	35.5	33.9	34.9	32.9	14.3	34.5	32.6	34.6	34.3	34.7	32.7	14.3
		33.8	32.9	33.8	35.4	33.8	32.4	14.3							
H9	Healthy	28.4	25.7	29.9	27.5	29.5	26.6	13.8	28.4	25.7	29.9	29.4	27.5	26.6	13.9
		28.4	25.7	29.9	27.4	29.2	26.7	13.9							
H11	Healthy	31.4	30.4	32.2	30.4	32.4	28.9	14.0	31.2	30.4	32.1	33.2	30.5	29.0	14.0
		30.9	30.3	32.0	30.6	34.0	29.0	14.0							
OA6	OA	26.7	29.6	29.6	28.5	27.5	27.5	14.0	26.8	29.6	29.6	27.3	28.6	27.5	14.0
		26.9	29.6	29.7	28.7	27.2	27.6	14.0							
OA8	OA	31.0	28.8	31.7	29.0	27.0	28.0	13.8	30.9	28.8	32.0	27.0	29.1	28.0	13.8
		30.9	28.8	32.3	29.2	27.1	27.9	13.8							
OA19	OA	28.0	30.4	30.3	29.1	27.5	27.3	14.2	27.9	30.3	30.2	27.3	29.2	27.4	14.0
		27.9	30.2	30.2	29.2	27.2	27.5	13.9							
OA28	OA	30.4	28.7	30.2	26.6	28.5	26.5	13.6	30.5	28.8	30.2	28.3	26.6	26.5	13.5
		30.5	28.9	30.3	26.6	28.2	26.5	13.4							
OA32	OA	34.7	35.3	34.5	33.5	29.8	32.7	13.6	34.7	35.3	34.4	29.9	34.5	32.5	13.5
		34.8	30.1	34.2	35.5	30.0	32.3	13.5							
OA35	OA	32.5	32.3	30.9	31.7	30.1	29.8	13.6	32.8	33.7	31.5	30.4	32.2	29.8	13.7
		33.1	35.1	32.1	32.8	30.6	29.7	13.7							
OA39	OA	33.9	31.0	31.3	31.9	31.6	27.8	13.7	33.6	31.4	31.5	31.6	31.5	27.8	13.7
		33.4	31.8	31.6	31.2	31.5	27.8	13.7							
OA44	OA	27.7	31.4	31.8	29.6	28.7	28.3	13.5	27.7	31.0	31.6	28.9	30.0	28.2	13.5
		27.8	30.7	31.3	30.4	29.0	28.1	13.6							
OA46	OA	33.6	31.9	ND	31.7	30.8	ND	13.6	33.9	31.7	ND	31.1	31.9	ND	13.6
		34.2	31.6	ND	32.0	31.3	ND	13.7							

OA47	OA	28.5	29.8	31.5	28.7	26.9	26.3	13.5	28.2	29.8	31.7	26.9	28.8	26.4	13.5
		27.9	29.7	31.8	28.8	26.9	26.4	13.6							
OA56	OA	26.6	26.6	27.9	27.3	22.5	26.5	13.4	26.6	26.6	27.9	22.6	27.2	26.7	13.5
		26.5	26.5	28.0	27.1	22.7	27.0	13.5							
OA58	OA	31.4	30.3	31.3	28.2	29.8	28.1	13.6	31.5	30.5	31.7	29.7	28.4	28.1	13.5
		31.5	30.6	32.1	28.5	29.5	28.2	13.5							
OA59	OA	32.2	30.1	32.2	33.9	29.0	29.8	13.9	32.0	30.2	32.4	29.1	35.0	29.8	13.9
		31.9	30.3	32.5	36.1	29.2	29.8	13.9							
OA61	OA	27.4	26.6	29.9	26.3	22.9	25.8	13.5	27.5	26.7	29.8	22.8	26.3	25.8	13.5
		27.6	26.7	29.6	26.3	22.8	25.8	13.6							

ND: no data
